# Impact of an accelerated melting of Greenland on malaria distribution over Africa

**DOI:** 10.1038/s41467-021-24134-4

**Published:** 2021-06-25

**Authors:** Alizée Chemison, Gilles Ramstein, Adrian M. Tompkins, Dimitri Defrance, Guigone Camus, Margaux Charra, Cyril Caminade

**Affiliations:** 1 Laboratoire des Sciences du Climat et de l’Environnnment (LSCE), CEA, Gif-sur-Yvette, France; 2 Abdus Salam International Centre for Theoretical Physics (ICTP), Trieste, Italy; 3 The Climate Data factory, Paris, France; 4 Department of Livestock and One Health, Institute of Infection, Veterinary and Ecological Sciences, University of Liverpool, Liverpool, UK; 5 Health Protection Research Unit in Emerging and Zoonotic Infections, University of Liverpool, Liverpool, UK

**Keywords:** Climate-change impacts, Malaria, Epidemiology

## Abstract

Studies about the impact of future climate change on diseases have mostly focused on standard Representative Concentration Pathway climate change scenarios. These scenarios do not account for the non-linear dynamics of the climate system. A rapid ice-sheet melting could occur, impacting climate and consequently societies. Here, we investigate the additional impact of a rapid ice-sheet melting of Greenland on climate and malaria transmission in Africa using several malaria models driven by Institute Pierre Simon Laplace climate simulations. Results reveal that our melting scenario could moderate the simulated increase in malaria risk over East Africa, due to cooling and drying effects, cause a largest decrease in malaria transmission risk over West Africa and drive malaria emergence in southern Africa associated with a significant southward shift of the African rain-belt. We argue that the effect of such ice-sheet melting should be investigated further in future public health and agriculture climate change risk assessments.

## Abstract (web summary)

Release of freshwater into the oceans as a result of ice sheet melting could impact the distribution of climate-sensitive diseases. Here, the authors show that a rapid ice sheet melting in Greenland could cause an emergence of malaria in Southern Africa whilst transmission risks in West Africa may decline.

## Introduction

Human malaria is a parasitic disease caused by five species of *Plasmodium* and is transmitted by the bite of an infected *Anopheles* female mosquito to a human host. The tropical form of the parasite, *Plasmodium falciparum*, causes the most severe clinical form of malaria and is widespread in Africa^1^. Malaria is responsible for many deaths worldwide, 405 000 reported in 2018, 67% occurred among children between 0 and 5 years of age^2^. Ninety-three percent of total cases and 94% of global deaths occurred in Africa in 2018^1^. Hence malaria has serious socio-economic impacts and can hamper development over the African continent^3^. Historically, the prevalence of malaria was significantly higher than today, even in temperate regions of Europe and North America^4^, before large control measures were undertaken following World War II^5^. Malaria elimination was achieved post 1970s for European countries and during the 1950s in the USA. Over the African continent, the situation started improving during the past two decades with increased financial support and malaria control efforts (insecticide spraying, distribution of bed nets, development of rapid diagnostic tests and drugs)^5^.

Malaria is a climate sensitive disease, with transmission often seasonal, as specific temperature and rainfall conditions are necessary for the development of *Anopheles* mosquitoes and *Plasmodium* parasites^6,7^. *An. gambiae, An. arabiensis* and *An. funestus* are the primary malaria vectors in the worst-affected regions of Africa^8^. They are present when humidity exceeds at least 40% but adult mosquitoes die rapidly above 38 °C^9^. Their presence is strongly regulated by the (often seasonal) rains, which are needed to provide breeding sites. After the sporogonic cycle, which is the incubation period for the mosquito to become infectious, the mosquito can infect additional humans. This incubation period shortens when temperature increases. A minimum temperature for sporogonic development was observed at 17 °C for *An. gambiae* and *P. falciparum*^10^. Therefore, temperatures must be high enough for the parasite to complete its sporogonic cycle but if the temperature is too high then the mortality of the vector increases, leading to a decrease in malaria transmission risk. It should be emphasized that the temperature thresholds and hydrological relationships are still studied and debated^10,11^. Thus, while control efforts and economical development have been successful in reducing transmission, changing climate conditions can still impact both the seasonality and mean intensity of malaria transmission^12^.

Several studies have investigated the impact of climate change on future malaria risk. Martens et al.^13^ indicate that climate change will mainly impact the fringes of endemic regions where malaria transmission occurs year-round. These fringe regions include South East Asia, South America, and parts of Africa^13^. Another study by the same author in 1999 indicates that temperate zones where competent *Anopheles* vectors are present but where the temperatures are too cold for transmission are the most at risk. These are North America, Central Asia, and northern Europe^14^. Martens also shows that the length of the Length of the transmission season (LTS) is impacted by changes in climate. Some regions, which are projected to be drier in the future, may experience a decrease in LTS. Several studies show that rising temperatures might lead to a decrease in malaria transmission in the warmest regions, as is the case in the semi-arid Sahel^15^. A temperature increase could, however, increase malaria transmission in colder regions such as the plateaus of East Africa^15^. These studies highlight the sensitivity of malaria transmission to changes in temperature and rainfall. Changes in the LTS were simulated for the twenty-first century by Caminade et al.^16^ using an ensemble of malaria and climate models driven by climate scenarios such as the Representative Concentration Pathway 8.5 (RCP8.5)^17^. This study highlights a decrease in LTS over the warm plains of West Africa and an increase in LTS over the plateaus of East Africa during the 2080s based on RCP8.5^16^.

One limitation of previous public health impact studies is that they have usually only considered standard climate change RCP scenarios produced within the Couple Model Intercomparison Project (CMIP) framework^18^. RCP span a range of greenhouse gases (GHGs) emission scenarios, but may neglect potential rapid climate destabilizing mechanisms, such as a rapid ice-sheet melting at high latitudes and accelerated melting of permafrost.

General Circulation Models (GCMs) are not usually fully coupled with ice-sheet models. Instead, their temperature and precipitation outputs are used to force ice-sheet models or regional models offline to estimate additional sea level rise (SLR)^19^. The melting of the ice sheet is not directly accounted for in the GCMs, nor is the effect of their feedbacks with the other sub-climatic systems. Melting is a nonlinear process due to freshwater inputs slowing down the Atlantic Meridional Overturning Circulation (AMOC)^20^ and due to positive feedbacks with temperature increase^19^, since the albedo of exposed surfaces decreases following the melting of the ice sheet leading to an increased absorbance and further melting of the ice sheet. In addition, the potential increase in liquid with respect to solid precipitation increases the mass loss of the Greenland ice sheet further^19^. There is also a mass loss due to glacier dynamics^19,21^. In Greenland, recent observations suggest that important processes responsible for glacier front destabilization that are not included in the current state of the art dynamical ice-sheet models^21,22^. Such destabilization could lead to an iceberg debacle similar to some extent to past Heinrich events^23^. While the IPCC fifth Assessment Report (AR5) predicts an additional SLR ranging between 0.52 and 0.98 m by the end of the twenty-first century^24^, rapid ice-sheet destabilization could significantly increase these estimates.

A significant release of freshwater at high latitudes would likely have significant consequences for climate over the European and African continents, directly affecting the AMOC^25,26^. Currently, the driving force behind the AMOC is the sinking of the cold, dense waters of the North Atlantic. This descent drives the ocean currents like a “conveyor belt”, allowing warm waters to rise from the south to the north and thus redistributing heat and water between hemispheres. An additional supply of freshwater in the Northern Atlantic would lead to a decrease in the density of cold surface water. This effect slows down the sinking of cold surface water and the AMOC. A slowdown of the AMOC affects the ocean temperature, as well as pressure gradients over the Atlantic ocean. The respective cooling of the North Atlantic and the Sahara desert with respect to the southern Atlantic ocean is usually associated with a southward shift of the rain-belt ultimately leading to decreased rainfall over the Sahel^26^.

Such extreme changes in rainfall over the African continent might have significant implications for both the location and intensity of malaria transmission that may fall outside the range predicted by standard RCP scenarios. As most previous impact studies have focused on standard climate change scenarios^27^, the purpose here is to investigate the impact of such a rapid ice-sheet destabilization of Greenland, on climate and malaria distribution in Africa using a multi malaria model approach. An ensemble of destabilization scenarios ranging from limited (SLR = 1 m) to extreme (SLR = 3 m) ice melt is utilized to estimate associated changes in climate and malaria risk over Africa and discuss potential public health implications. The aim of this study is not to make realistic projections of what will happen, but to use extreme cases of freshwater input to study mechanisms and patterns in one specific climate model.

## Results

### Malaria model validation and historical context

**Fig. 1:**
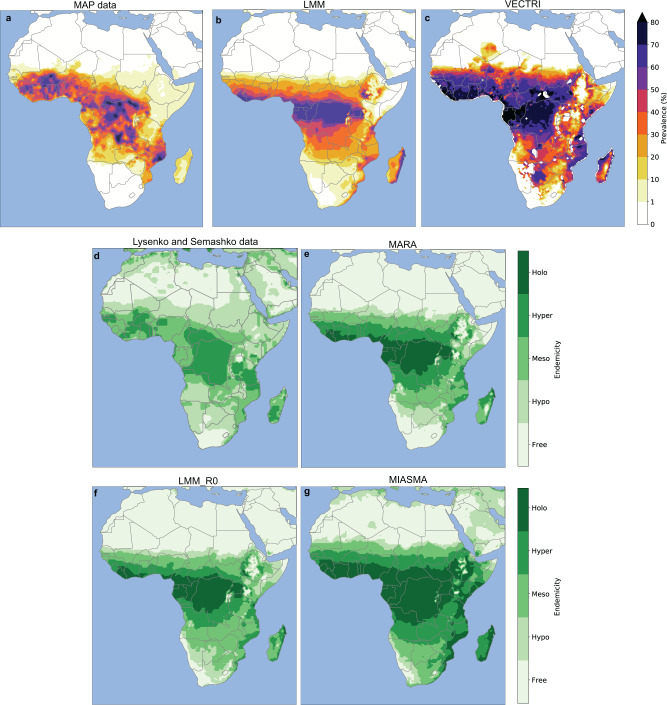
Malaria model validation maps. **a** Averaged MAP prevalence data for 2000–17. Averaged prevalence (%) for **b** LMM and **c** VECTRI. **d** Lysenko and Semashko malaria endemicity classes for 1900s. Endemicity classes for 1951 derived from **e** MARA, **f** LMM_*R*_0_ and **g** MIASMA.

Mean malaria prevalence (%), as simulated by Liverpool Malaria Model (LMM) (Fig. 1b) and VECtor borne disease community model of ICTP TRIeste (VECTRI) (Fig. 1c), is compared to Malaria Atlas Project (MAP) prevalence data (Fig. 1a) for 2000–2017.

Maximum MAP prevalence values reach about 70% over Central Africa and parts of West Africa (Fig. 1a). Prevalence hotspots are shown over Burkina Faso, parts of southern Guinea, the Democratic Republic of Congo, and parts of Mozambique. The surrounding regions still have large prevalence values ranging between 30% and 60%. Malaria prevalence values approach 0 over the north of the Sahel. Very low prevalence values are shown over the plateau of East Africa, the South of Namibia, Botswana, and South Africa (Fig. 1a).

The malaria-free zones are well captured by the LMM except over Ethiopia that is considered malaria-free by the MAP data (Fig. 1a, b). The northern malaria fringe south of the Sahara is simulated too far south by the LMM model (Fig. 1b) with respect to MAP data (Fig. 1a). The largest prevalence values are shown over Western and Central Africa. LMM simulated prevalence is distributed zonally, namely prevalence linearly decreases with increasing or decreasing latitude from the equator (Fig. 1b). The MAP data show a more heterogeneous prevalence pattern with localized maximum shown over West, Central, and South-Eastern Africa (Fig. 1a). VECTRI model overestimates prevalence compared to MAP and LMM (Fig. 1a–c). VECTRI shows prevalence hotspots (>70%) over the same regions as MAP but these hotspots encompass a much broader geographical range (Fig. 1c). The malaria-free regions south of the Sahara are reproduced at the right latitude by the VECTRI model but it simulates malaria risk over the southern part of Algeria, this feature is absent in MAP or LMM simulations. Eastern and southern African regions which are normally malaria-free, reach prevalence values >60% in VECTRI simulation (Fig. 1c). It is nonetheless noteworthy that MAP data represent recent malaria distribution and includes the effect of interventions, which is not considered in LMM and VECTRI. The spatial range of malaria was far greater during the pre-intervention era, as represented by the Lysenko and Semashko data, and the VECTRI model is closer to this early twentieth century estimate.

**Fig. 2:**
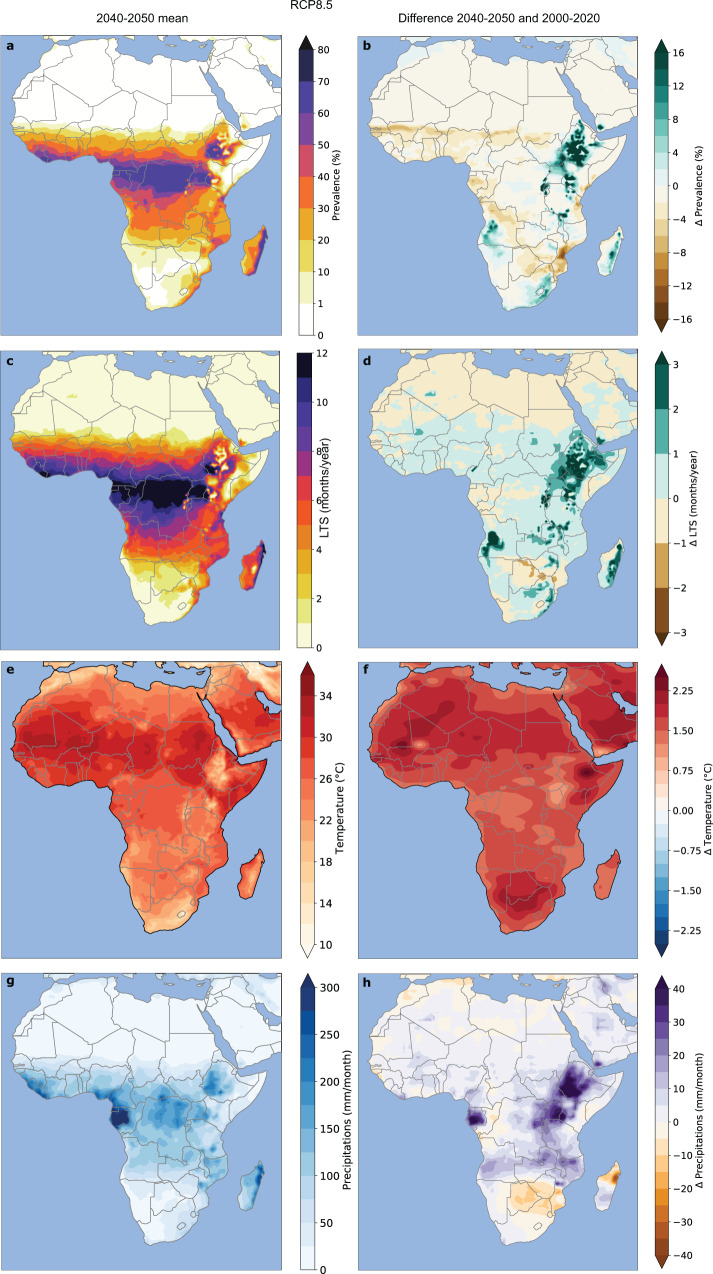
Mean and future changes in climate parameters and malaria risk based on the RCP8.5 scenario. The left-hand column (**a**–**c–e**–**g**) depicts mean patterns for the 2040s and the right-hand column (**b**–**d**–**f**–**h**) shows future differences between the 2040s and 2010s. **a**, **b** malaria prevalence (%) simulated by the LMM, **c**, **d** LTS (in months) based on the MARA model. **e**, **f** temperature (°C) and **g**, **h** precipitation (mm.month^−1^).

All Mathematical Malaria Models (MMM) are solely driven by climate variables, and maximum in prevalence are simulated over Central Africa (Fig. 1), where the largest rainfall occurs (Fig. 2g). The LMM and VECTRI malaria-free areas correspond to regions where climate is unsuitable for malaria transmission and where population densities are extremely low. The MAP maximum mostly corresponds to regions where the *Anopheles* mosquitoes became resistant to insecticides. Our ensemble of MMMs does not consider the effect of control and socio-economics factors which is considered in MAP.

Simulated LTS was transformed into endemicity classes (see “Methods”) and compared to the Lysenko and Semashko data^4^ (Fig. 1d–g), to validate the monthly MMMs, the Mapping Malaria Risk in Africa (MARA) model, Modeling Framework for the Health Impact Assessment of Man-Induced Atmospheric Changes (MIASMA) and the steady state version of LMM (LMM_R_0_). The holoendemic area is shown over Central Africa, especially over the Democratic Republic of Congo (Fig. 1d). Patchy maxima are shown over coastal West Africa and Burkina Faso (Fig. 1d). Holoendemic transmission is also shown over Tanzania (Fig. 1d). Around those regions, hyperendemic transmission is depicted from West to Southeast Africa (Fig. 1d).

Over the northern fringe of the Sahel and southern Africa, malaria transmission is mostly mesoendemic (Fig. 1d–g). The Sahara Desert, parts of South Africa and high plateaus of East Africa were malaria-free during the 1900s (Fig. 1d). Notably, meso to hyperendemic malaria transmission is also shown over the coasts of North Africa for the Lysenko and Semashko data, and this feature is not well captured by the MMMs (Fig. 1d–g). This is consistent with the fact that the Lysenko and Semashko data are based on all *Plasmodium* species (including the more temperate form of the parasite, *Plasmodium vivax*, that used to be prevalent over North Africa), while our MMMs are solely parameterized for the transmission of the tropical form of the parasite, *P. falciparum*.

The MARA and LMM_R_0_ models provide similar results (Fig. 1e–f), and they are both in good agreement with the Lysenko and Semashko data (Fig. 1d). The holoendemic regions encompass West and Central Africa. Near those areas, malaria endemicity varies from hyperendemic to mesoendemic. Hypoendemic regions are distributed from North Africa to the South of the Sahara, on the East African plateaus, and for LMM_R_0_ over the southern part of South Africa. The MARA model simulates moderate transmission over the northern part of South Africa e.g. over the Limpopo region and Krueger park where moderate malaria transmission still occurs today. The hyperendemic zones, as simulated by MIASMA (Fig. 1g), cover a broader geographical range with respect to other MMMs and the Lysenko and Semashko data (Fig. 1d–f). These zones are distributed from East to West Africa, between 14°N and 17°S. Around those areas, malaria transmission decreases, switching from wide hyperendemic zones to smaller mesoendemic zones. Hypoendemic regions are mainly restricted to the Sahara, to part of the Namibian coasts, and to high altitude regions of East Africa.

Overall, MARA, LMM_R_0_, and LMM correctly simulate malaria transmission at regional scale, while the MIASMA and VECTRI models tend to overestimate malaria transmission and its geographical range. Different validation scores are provided in Table 1: the Pearson correlation test, the Normalized Mean Absolute Error (NMAE), and the Root Mean Square Error (RMSE) were calculated for each model. The largest correlation coefficient is shown for LMM prevalence (0.527) and MARA LTS (0.561). The LMM model and MARA models also show better RMSE and NMAE scores. MMMs differ in their application and therefore in their formulation. The threshold values for temperature and precipitation vary between each MMM (see “Methods”). Therefore, MMMs do not have the same sensitivity to changes in rainfall and temperature (Supplementary Fig. 1). In addition, the outputs of the monthly MMMs differ, they are transformed to obtain the LTS using different assumptions, and this can lead to large differences despite identical climate inputs. In the following, we will primarily focus on the two best performing models (LMM and MARA), except for the innovative question of the additional ice-melting effect where the 5 models are presented. Results for other MMMs are presented in Supplementary Information.

**Table 1:** Malaria models skill scores.

	LMM (Prevalence)	VECTRI (Prevalence)	MARA (LTS)	MIASMA (LTS)	LMM_R_0_ (LTS)
Pearson test	0.527*	0.404*	0.561*	0.441*	0.501*
NMAE	0.127	0.149	0.193	0.381	0.227
RMSE	0.185	0.228	0.011	0.016	0.012

Different skill scores (Pearson correlation coefficients, NMAE and RMSE) were calculated.

The MAP prevalence data are used as baseline for LMM and VECTRI, and the Lysenko and Semashko data are used as baseline for MARA, MIASMA, and LMM_R0. Significant correlations at the 99% confidence interval are denoted by *.

*LTS* Length of the Transmission Season, *NMAE* Normalized Mean Absolute Error, *RMSE* Root Mean Square Error.

### Impact of RCP8.5 simulation on malaria

The largest increases, in simulated prevalence (Fig. 2b) and LTS (Fig. 2d), are shown over the plateaus of East Africa for the RCP8.5 scenario. Maximum values slightly exceed 60% for simulated prevalence (Fig. 2a) and LTS can sometimes exceed 10 months (Fig. 2c), denoting almost year-round malaria transmission.

These maxima are shown in areas where temperatures typically range from 22 to 30 °C (Fig. 2e). The highest rainfall amounts ranging from 100 to 300 mm.month^−1^ are simulated over the western coast of Central Africa (Fig. 2g). The areas free of malaria coincide with temperatures above 32 °C or below 20 °C and arid areas (below 50 mm.month^−1^, Fig. 2e–g).

All MMMs simulate an increase in LTS and malaria prevalence over the highlands of East Africa during the 2040s (Fig. 2b–d and Supplementary Fig. 2) for the RCP8.5 scenario. The simulated increase in prevalence reaches about 18% and the future LTS is simulated to lengthen by about 3 months over Ethiopia (Fig. 2b–d). This increase is associated with a rise in temperature of up to +3 °C (Fig. 2f) and an increase in precipitation of at least 40 mm.month^−1^ (Fig. 2h). An increase in prevalence and LTS is also shown over the plateau of central Angola (Fig. 2b–d), this simulated increase is consistent with an increase in temperature and rainfall (Fig. 2f–h). Conversely, the dynamical LMM model simulates a decrease in prevalence over the Sahel and southern Africa (Fig. 2b). These differences are associated with a 1 °C temperature increase over the Sahel (Fig. 2f) where annual mean temperatures were already exceeding 30 °C (Fig. 2e). However, there is no major change in simulated rainfall over this region (Fig. 2h). These results are in agreement with former findings based on a larger multi-model risk assessment^16,28^. In addition, the simulated decrease in malaria risk over the plains of West Africa and the increase shown over the plateaus of East Africa are similar to twentieth century malaria endemicity trends (1900–2007)^29^.

If we consider the standard RCP8.5 scenario, rising temperatures tend to moderately shorten the length of the future LTS over the plains of the Sahel (Fig. 2b–d). Over these regions, future temperatures might become too warm for *Anopheles* mosquitoes to survive. In contrast, there is a simulated increase in the LTS shown over the East African highlands (Fig. 2b–d). Future temperature exceeds the minimum temperature threshold for the parasite to replicate inside the mosquito vector. This effect is accentuated by a simulated increase in precipitation over this region (Fig. 2h).

### Additional impact of a rapid ice-sheet melting on malaria

**Fig. 3:**
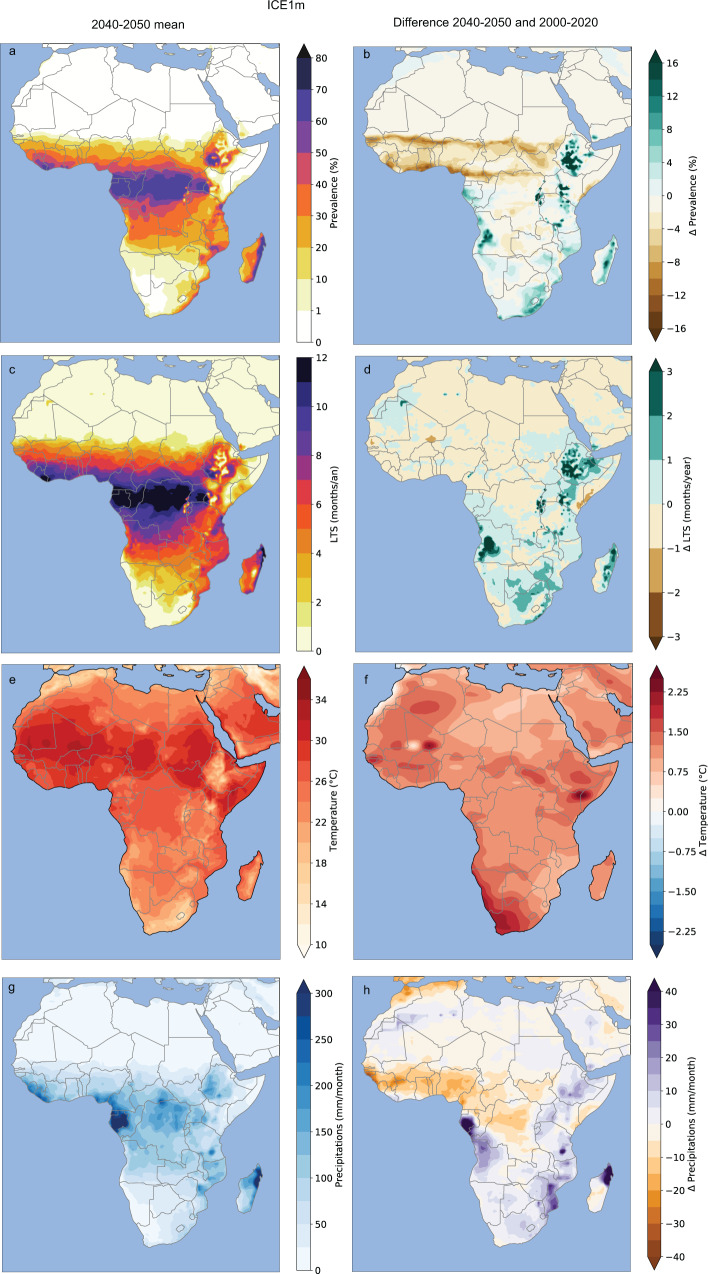
Mean and future changes in climate parameters and malaria risk for the ICE1m experiment. The left-hand column (**a**–**c**–**e**–**g**) depicts mean patterns for the 2040s and the right-hand column (**b**–**d**–**f**–**h**) shows future differences between the 2040s and 2010s. **a**, **b** Simulated malaria prevalence (%) by the LMM. **c**, **d** LTS (in months) based on the MARA model. **e**, **f** Temperature (°C) and **g**, **h** precipitation (mm.month^−1^).

In the ICE1m experiment, the simulated temperature increase is more moderate with respect to the RCP8.5 simulation (Fig. 3f vs Fig. 2f). However, simulated rainfall change in ICE1m significantly differs from RCP8.5, with a significant rainfall decrease shown over the northern half of the African continent, while an increase in rainfall is simulated over southern Africa, denoting a southward shift of the African rain-belt (Fig. 3h). There is a strong increase in both the LTS and malaria prevalence over the southern part of Africa and over the Eastern African plateaus (Fig. 3b–d). A clear decrease in simulated malaria prevalence is depicted over south of the Sahara with the LMM model (Fig. 3b). The same analysis is presented in Supplementary Information for the other MMMs (VECTRI, MIASMA, and LMM_R_0_, see Supplementary Figs. 2 and 3) and for other ICEXm simulations (ICE0.5m, ICE1.5m, ICE3m, see Supplementary Fig. 6).

**Fig. 4:**
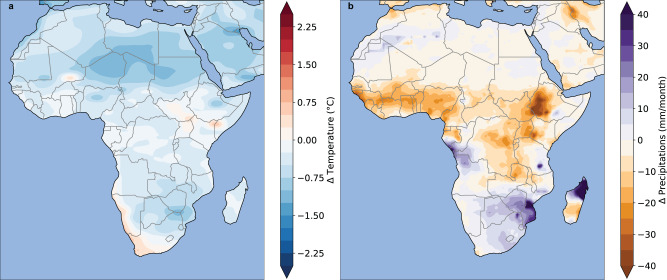
Impacts of an additional ice-melting on climate parameters. **a** Temperature (°**C**) and (**b**) rainfall (mm.month^−1^) difference between the ICE1m and RCP8.5 simulation for the 2040s.

**Fig. 5:**
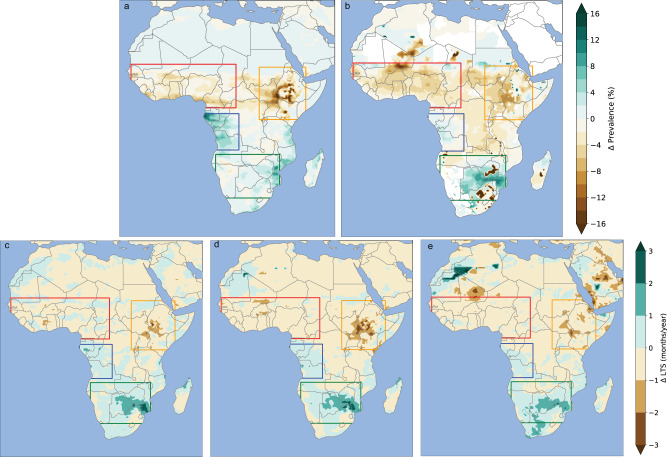
Difference in malaria risk between the ICE1m simulation and the RCP8.5 simulation for the 2040s. Difference in prevalence (%) for (**a**) LMM and (**b**) VECTRI and difference in LTS (months) for (**c**) LMM_R_0_, (**d**) MARA, and (**e**) MIASMA. The different boxes represent key Africa regions (red for West Africa, orange for East Africa, blue for west central coasts of Africa, green for southern Africa), see “Methods” for further details.

In order to focus on the additional effect of the ice-melting signal, the difference between the ICE1m and the RCP8.5 simulation for temperature and precipitation is shown in Fig. 4, while changes in malaria transmission are shown in Fig. 5. Simulated temperatures tend to be colder in ICE1m with respect to RCP8.5 over Africa, except over the western coast of Namibia and the southern-east coast of Africa where a local increase in temperature is simulated (Fig. 4a). Simulated temperatures are colder over the Sahara Desert and to a lesser extent over southern Africa, in ICE1m with respect to RCP8.5 (Fig. 4a). A very pronounced drying signal is simulated over the Sahel, East Africa, and Central Africa whereas a significant increase in rainfall is simulated over southern Africa (Fig. 4b). These climatic changes have an impact on malaria prevalence in all MMMs simulations (Fig. 5).

The rapid melting of Greenland tends to reduce malaria transmission further over the Sahel. This decrease is moderate for ICE1m (Fig. 5c–e) and intensifies in ICE3m (Supplementary Fig. 7). The simulated difference in malaria prevalence ranges between −4 to −8% for VECTRI and LMM over the Sahel (Fig. 5a, b). This decrease is more pronounced in the VECTRI experiment (Fig. 5b) with respect to the LMM simulation (Fig. 5a). The simulated increase in malaria transmission shown in RCP8.5 is reduced in ICE1m over the plateaus of East Africa (Fig. 5). For LMM this signal ranges from −6 to −16% (Fig. 5a) while for VECTRI this signal does not exceed −10% (Fig. 5b). For LMM_R_0_ and MIASMA, LTS shortens by about one month between ICE1m and RCP8.5 (Fig. 5c–e). MARA simulates a LTS difference ranging between −1 and −3 months over the plateaus of East Africa (Fig. 5d). The additional impact of ice-sheet melting tends to increase malaria transmission risk over the western coasts of Central and southern Africa (Fig. 5). This signal ranges between 6 and 14% for simulated malaria prevalence and between 1 and 3 months for simulated LTS over southern Africa at the end of the twenty-first century". (Supplementary Fig. 4).

Based on these future changes, four geographical areas were defined to study the temporal evolution of malaria transmission and its relationship with simulated changes in temperature and rainfall (Supplementary Fig. 4.a for malaria parameters and Supplementary Fig. 4.b for climatic parameters). The largest changes are simulated over southern Africa at the end of the twenty-first century and are related to an increase in precipitation (Supplementary Fig. 4.4). Changes in endemicity classes are provided in Supplementary Table 1 and Supplementary Fig. 5. For more than 80% of spatial points there is no change in endemicity classes (Supplementary Table 1). For the remaining points, endemicity classes change by one class maximum.

## Discussion

Malaria transmission risk increases during the twenty-first century over highland regions of East Africa while it slightly decreases over West Africa based on the RCP8.5 scenario. The decrease in the West is mainly due to temperatures exceeding the upper survival threshold of *Anopheles* mosquitoes, while the temperature increase over the East African plateaus exceeds the minimum sporogonic threshold. By including an additional freshwater release in the North Atlantic Ocean, climate and malaria risk are significantly modified. These changes are consistent across all malaria models. The increase in risk over East Africa is more moderate due to lower temperature and precipitation conditions with respect to the RCP8.5 scenario. A rapid ice melting induces a southward shift of the African rain-belt. This latitudinal shift amplifies the simulated decrease in malaria risk over West Africa and increases the risk over southern Africa. This could have serious implications for the populations of southern Africa, which have not been intensively exposed to malaria in recent decades.

If we consider the current state of the art global and regional climate models, the African monsoon is a complex phenomenon to represent and simulate correctly. First, there is a strong natural decadal to multi-decadal variability in African rainfall^30^. For example, mega-droughts occurred over the Sahel during the early nineteenth century, before the Anthropocene took place^31^. Second, the response of the African monsoon to different RCP emission scenarios has been summarized in the WG1 report of the IPCC AR5 report. Results show large differences in simulated rainfall by different GCMs over the twenty-first century^32^. For instance, simulated rainfall changes in the 32 GCM ensemble for RCP8.5 are quite diverse, some models show an enhanced monsoon, while others simulate a drying signal. Some climate models depict a latitudinal shift of the rain-belt whereas others models only show changes in rainfall intensity with no clear latitudinal shift of the monsoon. Although there is a strong variability between models regarding future climate change with standard RCP emission scenarios, the southward shift of the African monsoon due to freshwater release is a robust result. During past glacial periods, rapid Northern ice-sheet melting led to global reorganization of ocean and atmosphere dynamics. Over North Atlantic, the surge of icebergs and associated freshwater triggered a decrease of North Atlantic Deep Water and a slowdown of the AMOC. Over Africa, in the tropics, the atmospheric circulation response corresponds to a large subtropical jet variation and a southward shift of Inter-Tropical Convergence Zone (ITCZ)^33^. This change is recorded in paleo proxy of temperature and precipitation^34^ and confirmed by most climate model simulations ran in paleo mode^35^. The decrease of the AMOC is triggered by freshwater inputs in our simulations. Such a large decrease of the AMOC and its stability may be produced using GHGs solely^20,36^. Indeed, the synergistic contribution of Greenland ice-sheet melting (freshwater release) and GHGs warming may lead to a collapse of the AMOC^20^. In addition, very recent studies show that there is a current slowdown of AMOC leading to the weakest state of the AMOC occurring in recent decades^37^. A southern shift of the ITCZ and subtropical jet shifts the African monsoon southward^26,38^.

Simulated changes in malaria transmission based on the RCP8.5 scenario are consistent with other studies^28,39^. Nevertheless, the MMMs used in this study have significant limitations. Only the direct impact of climate is considered in our malaria models, except for the VECTRI model which includes population density effects which are fixed in time. Our models do not consider age stratification and dynamic population changes^2^ and do not consider changes in control measures, the development of new malaria drugs and treatments, and importantly changes in socio-economic factors that significantly impact and shape malaria burden at country scale^5^. Moreover, the VECTRI and LMM models are validated by a statistical model (MAP) which itself can be biased. On the other hand, the IPSL climate model does not account for all climate change-related feedbacks. Indeed, dynamical changes in temperature and precipitation inevitably affect vegetation, which in turn could affect mosquito dynamics at small spatial scale^40,41^. Finally, an increase in spatial resolution is necessary to take into account the hydrology of basins (with the presence of river, lakes...) and dams^42^. In addition, we only use one climate model for practical reasons. Running several climate models with a freshwater release is a time-consuming exercise and will require several international collaborations with other climate centers worldwide. Such “tipping point” multi-climate model exercise should be encouraged in the future.

Our scenario shows the importance of the freshwater release in the North Atlantic in terms of feedback on temperature and precipitation and therefore on malaria transmission and potentially other climate-sensitive diseases. Given the accelerated pace of observed environmental changes, particularly over peri-Arctic and Arctic regions, it is now critical to develop novel climate scenarios which consider such rapid climate tipping points. Assessing the impact of such tipping point scenarios (including a rapid ice-sheet melting at high latitudes, but also permafrost melt) and the associated uncertainties on critical sectors, such as public health, agriculture, and water resources, should be a research priority for the climate and impact modeling communities in the upcoming years.

## Method

### Malaria models

An ensemble of five MMM is used. Two MMMs, the LMM^43^ and the VECTRI^44^ are dynamical malaria models that simulate the vector and parasite dynamics, and they are both driven by daily rainfall and temperature data. The other three MMMs are simpler in their formulation and are driven by monthly rainfall and temperature data. These models are the MARA model^45^, MIASMA^46^, and the steady state version of LMM (LMM_R_0_). All malaria models have been parameterized to simulate the transmission of *P. falciparum*, the malaria parasite which causes about 90% of all plasmodial infections in Africa^1,47^. It is noteworthy that most MMMs have been parameterized based on field and laboratory data for *An. gambiae*. For the three models driven by monthly climate data, the simulated LTS (number of months per year) is used for model intercomparison. For the two dynamical MMMs, we investigate malaria prevalence (in %) which is the proportion of the human population infected with malaria.

The MARA model is a MMM that estimates the distribution of stable malaria transmission^45^. This model simulates the effect of monthly mean rainfall and temperature on malaria transmission. This model requires three consecutive months with more than 60 mm of rain combined with a catalyst month with more than 80 mm. The temperature must be higher than 19.5 °C, and a temperature function is combined with the seasonality index derived from the standard deviation of monthly rainfall^16^. The resulting output for a given month is 1 if climate is suitable for malaria transmission and 0 otherwise^45^. LTS is then calculated as the sum of the number of months (consecutive or not) suitable for transmission for a given year. This model has originally been developed to estimate climate suitability for malaria at the African continental scale and does not consider micro-climatic conditions, watershed specificity, and the impact of human intervention^45^.

MIASMA is a dynamical monthly malaria model^14^. MIASMA is based on simple functions linking monthly mean temperature and precipitation to malaria Transmission Potential (TP). Temperature affects the probability of survival and mosquito biting rate, and a minimum of monthly precipitation is essential for malaria transmission^14^. The temperature range suitable for potential transmission ranges from 16 to 40 °C. In addition, rainfall must be greater than or equal to 80 mm.month^−1^ for 4 consecutive months^14^. If TP > 0 (equivalent to a basic reproduction number *R*_0_ > 1), we consider that a given month is suitable for transmission. LTS is then calculated by summing all suitable months (consecutive or not) for a given year.

LMM is an epidemiological and dynamical malaria model. It simulates the dependency of the malaria vector and parasite to daily rainfall and temperature conditions^12,43,48^. This model provides information for both *Anopheles* vector and the human host. It classifies the vector and host population into three categories susceptible, exposed, and infectious. Precipitation during the previous 10 days influences the number of mosquito eggs and the mortality of the larvae. Temperature influences the mortality rate of the mosquito, the egg-laying/biting (gonotrophic) cycle, and the incubation time of the parasite in the mosquito (sporogonic cycle). Both cycles depend on the number of degree days above a specific temperature threshold. For the gonotrophic cycle, the threshold is 9 °C and takes about 37 humid degree days above 7.7 °C, whereas the sporogonic cycle takes approximately 111 degree days with a threshold of 18 °C^49^. LMM has many epidemiological parameters as outputs. We focus on malaria prevalence (in %) which is the infected population rate.

LMM_R_0_ is a simplified, steady-state version of LMM^12^. This model uses monthly rainfall and temperature data as inputs. Rainfall influences the number of adult mosquitoes for each month. The minimum temperature threshold is 18 °C. The probability of surviving mosquitoes is a function of temperature, with a near-zero probability (0.04) for a temperature of 40 °C^12^. The output is the basic reproduction number (*R*_0_), which indicates the number of secondary infections arising from one infected case in a totally susceptible population. For a given month, if *R*_0_ > 1 then we assume that climatic conditions are suitable for malaria transmission and we assign 1 for this particular month and location. LTS is then calculated as the sum of suitable months (consecutive or not) for a given year.

VECTRI is a mathematical model that includes the effects of precipitation, temperature, population immunity, hydrology (using a simple pool model), and human population density^44^. VECTRI has been applied to both climate change scenarios^16^ and seasonal prediction of malaria. The inputs are daily precipitation, daily temperature, and population density. This model considers the impact of temperature and precipitation variations on the life cycle of the vector (larvae and adults) and the *P. falciparum* parasite similarly to LMM. The model parameterization is mostly based on data available for *An. gambiae*. The temperature affects both sporogonic and gonotrophic cycles. A hydrological basin model also represents the precipitation effect. The number of breeding sites increases following rainfall events and then decreases when evaporation and runoff increase. Extreme rainfall limits the number of larvae through a flushing effect^44^. As the entomological inoculation rate increases, it drives increasing immunity in the population following the model of Laneri et al.^50^, which is lost over a 3-year timescale, and immune populations are subject to lower transmission probabilities related to the susceptible population. VECTRI also considers population density when calculating the vector-biting rate. The population density used is that of the Afripop project for the year 2005^51^. Large population density leads to a dilution effect, leading to lower transmission in urban environments. While the base values of the model parameter settings are taken from published laboratory and field studies, these parameters are then calibrated within a set tolerance range (also estimated from the literature if available) using a constrained genetic algorithm machine learning approach described by Tompkins, A. & Thomson, M.^52^. Fifteen model parameters were calibrated fitting the model to over 200 field studies reporting the parasite ratio spanning the period 1980–2012 for a wide range of lowland and highland locations across Ethiopia, available from the Oxford MAP database^53^. The VECTRI malaria model has many standard outputs^44^. We focus on simulated malaria prevalence (in %) to compare VECTRI with LMM results.

### Malaria validation data

In order to validate the malaria model simulations, we employ two historical malaria datasets (Fig. 1). The first dataset is malaria prevalence in children at 2–10 years of age (in %) derived from the MAP^53^. MAP provides gold standard datasets for spatial malaria risk estimate. The MAP output data are based on a Bayesian statistical model, which is driven by a set of environmental and other socio-economic covariates^54^. Simulated malaria prevalence rates by VECTRI and LMM are directly compared to the MAP annual data. The period used for validation corresponds to the average between 2000 and 2017. The regions where prevalence values are below 1% are considered free of malaria risk.

The second dataset is based on the work conducted by Lysenko and Semashko during the 1960s^4^. This dataset corresponds to malaria prevalence data for children (PR) data that was transformed into malaria endemicity categories. These data provide 1900s malaria endemicity estimates for all *Plasmodium* parasites before human intervention occurred at a global scale. This dataset is a better estimate of the impact of environmental conditions on malaria burden, as limited malaria control measures were undertaken prior to the 1920s^55^. These data are binned into five categories: free, PR < 1%; hypoendemic, PR > 1% and PR < 10% ; mesoendemic, PR > 10% and < 50%; hyperendemic, PR > 50% and < 75%; and holoendemic transmission, PR > 75%^56^. The monthly malaria models (MARA, MIASMA, and LMM_R_0_) are compared to the Lysenko and Semashko data. The LTS malaria model output was transformed in endemicity categories, holoendemic transmission was defined for LTS > 9 months, hyperendemic transmission for LTS ranging between 6 and 9 months, mesoendemic for LTS ranging between 3 and 6 months, hypoendemic for LTS ranging between 1 and 3 months and free for LTS < 1 month^14^.

There is a 51-year gap between the Lysenko and Semashko data (1900) and our simulation data that starts in 1951. The difference between the 1900s and 1950s for malaria transmission is mostly related to the implementation of health measures and land surface management such as implementation of drainage schemes. However, our models do not take human intervention into account, which is why we assume that the Lysenko and Semashko data and the simulations of our models are comparable.

### Climate model and simulations experiments

All MMMs require temperature and rainfall data as inputs. To obtain these variables for different ice melt scenarios, we utilize General Circulation Model (GCM) simulations performed with the Institute Pierre Simon Laplace Climate Model at Low spatial Resolution, version A (IPSL-CM5A-LR)^57^. IPSL-CM5A-LR is part of the fifth phase of the Coupled Model Intercomparison Project (CMIP5)^58^, that underpins the IPCC AR5. It is a standard climate model that couples an atmosphere-land surface model to an ocean-sea ice model. This global model is made up of physical and biogeochemistry models^57^. The physical model for the atmosphere is the LMDZ model (Laboratoire de Météorologie Dynamique Zoom) in its version 5A^59^. It comprises 39 vertical levels with 15 levels below 20 km of altitude^57^. The land surface model is ORCHIDEE (ORganizing Carbon and Hydrology In Dynamic EcosystEms)^60^. The ocean model is NEMOv3.2 (Nucleus for European Modeling of Ocean)^61^. It includes OPA (Océan PArallélisé) to simulate the dynamics of oceans^57^, PISCES (Pelagic Interaction Scheme for Carbon and Ecosystem Studies) for ocean biochemistry^62^, and LIM2 (Louvain-la-Neuve Sea Ice Model, Version 2) for sea ice^63^. The OAsis (Ocean Atmosphere Sea Ice Soil) coupler^64^ allows the synchronization of all models and the exchange of energy and moisture fluxes between the different sub-climatic systems^57^. The biogeochemistry models are INCA (The INteraction with Chemistry and Aerosol) for tropospheric chemistry and aerosols^57^, the REPROBUS (Reactive Processes Ruling the Ozone Budget in the Stratosphere) module for stratospheric chemistry^65^. The prescribed variables are CO_2_ emissions based on RCP scenarios^17^, land use^66^, solar irradiance^67^, other gases emissions, and volcanic aerosols^57^. The spatial resolution of IPSL-CM5A-LR is 3.75° in longitude and 1.875° in latitude^57^.

Our reference simulation is the RCP8.5 simulation^17^ that was carried out with the same climate model. This experiment is a worst-case scenario, with GHGs emissions projected to result is a top of atmosphere radiative imbalance of 8.5 W m^−2^ by the end of the twenty-first century. It assumes GHG emissions approximately follow the current trend in future without mitigation^17^.

The second ensemble of simulations (ICEXm) represents the additional impact of ice melt. ICEXm simulations include the same RCP8.5 external forcing to which is added an accelerated melting of Greenland. ICEXm simulations correspond to a water-hosing experiment, which is superimposed on the standard RCP8.5 climate change scenario^26^. These simulations are referred as to ICEXm, where Xm denotes the additional SLR (in meters) at a global scale. In ICE1m an additional 0.22-Sv (1*Sv* = 10^6^ m^3^ s^−1^) of freshwater is released in the North Atlantic Ocean from 2020 to 2070^26^. ICE1m does not represent the total melting of Greenland which would correspond to an additional 7.2 m SLR, but is consistent with the maximum SLR estimate provided by the IPCC. Other ICEXm scenarios have been used and are presented in Supplementary Information. They respectively represent 0.5, 1.5, and 3 m of additional SLR. The ICE3m simulation corresponds to a significant destabilization of the ice sheet, which is similar to past Heinrich events.

Because of the complexity of modifying several climate models to simulate the melting of the ice sheet and computational time constrains, we have chosen to use only one climate model (IPSL-CM5A-LR) in this study.

Because GCMs still have significant biases in rainfall and temperature, in particular over Africa^68^, we employed a bias-correction technique to calibrate the raw GCM outputs. We used the Cumulative Distribution Function transform (CDF-t) method that was developed by Michelangeli et al.^69^. This method consists in matching the CDF of a simulated climate variable (rainfall and temperature outputs of the IPSL-CM5A-LR model) to the CDF of an observed climate variable through a mathematical transfer function^70^. The CDF-t method has been applied over the period 1950–2099. The observed reference dataset used for bias correction is WFDEI (Watch Forcing Data by making use of the ERA-Interim)^71^, which is derived from the ERA-Interim reanalysis data^72^, and available from 1 January 1979 to 31 December 2013. The CDF-t technique has been applied monthly to consider strong seasonality in rainfall and temperature in Africa. The CDF-t bias-calibration technique preserves the long-term trends but moments or quantiles are not conserved^70^. The GCM data has been spatially interpolated to match the WFDEI 0.5 × 0.5 degree grid before applying bias correction^71^.

We primarily focus on two simulations, the RCP8.5 simulation and the ICE1m simulation. The reference historical period is the average between 2000 and 2020 (referred as to 2010s). The maximum disturbance period was determined to occur between 2040 and 2050 (2040s) consistently with Defrance et al.^26^. Thus, difference maps were calculated as the difference between the maximum perturbation period (2040s) and the reference period (2010s). To estimate the additional impact of a rapid ice-melting, the difference between the ICE1m and RCP8.5 experiments were investigated for the 2040s. To focus on changes in seasonality, we carry out a time series analysis for four specific regions chosen due to their large malaria signals simulated during the 2040s. These regions are defined as follows: the West African region [2.1–15.1°N; 16.1°W to 20.1°E], the East African region [2.1°S to 16.1°N; 28.1–41.1°E], the Central coasts of Africa [12.6°S to 0.1°N; 9.1°E to 21.1°E] and southern Africa [28.1–12.6°S; 13.1–35.1°E]. Time series were calculated by averaging land values. A 6-year running average was applied to all time series and relative changes were calculated in percentages.

### Reporting summary

Further information on research design is available in the Nature Research Reporting Summary linked to this article.

## Supplementary information

Supplementary Information: Impact of an accelerated melting of Greenland on malaria distribution over Africa

Reporting Summary

## Data Availability

All model inputs and outputs that support the findings of this study are available at 10.17605/OSF.IO/RHKPB that should allow reproductibility of the main figures. The MAP data can be accessed by following this link: https://malariaatlas.org/explorer/#, they are available for use under the Creative Commons Attribution 3.0 Unported license. The Lysenko and Semashko data are the digitized data from the paper of Gething et al.^29^.

## Code availability

The code of the VECTRI model is available in open-source at the following link: http://users.ictp.it/~tompkins/vectri/. For other models, please contact the creators of these codes: Anne Jones (anne.jones@ibm.com) or Andy Morse (a.p.morse@liverpool.ac.uk) for LMM and LMM_*R*_0_—M. Craig for MARA (craigm@mrc.ac.za) and Pim Martens for MIASMA (p.martens@icis.unimaas.nl).

## References

[1] World Health Organization. World Malaria Report 2019 (WHO, 2019).

[2] Griffin JT, Ferguson NM, Ghani AC (2014). Estimates of the changing age-burden of Plasmodium falciparum malaria disease in sub-Saharan Africa. Nat. Commun..

[3] Chima RI, Goodman CA, Mills A (2003). The economic impact of malaria in Africa: a critical review of the evidence. Health policy.

[4] Lysenko, A. J. & Semashko, I. N. in Itogi Nauki: Medicinskaja Geografija (ed. Lebedew, A. W.) 25–146 (Academy of Sciences, Moscow, 1968).

[5] Feachem RG (2010). Shrinking the malaria map: progress and prospects. Lancet.

[6] Paaijmans KP, Blanford S, Chan BH, Thomas MB (2012). Warmer temperatures reduce the vectorial capacity of malaria mosquitoes. Biol. Lett..

[7] Bayoh MN, Lindsay SW (2003). Effect of temperature on the development of the aquatic stages of Anopheles gambiae sensu stricto (Diptera: Culicidae). Bull. Entomol. Res..

[8] Sinka ME (2012). A global map of dominant malaria vectors. Parasites Vectors.

[9] Kreppel K (2019). Impact of ENSO 2016–17 on regional climate and malaria vector dynamics in Tanzania. Environ. Res. Lett..

[10] Waite JL, Suh E, Lynch PA, Thomas MB (2019). Exploring the lower thermal limits for development of the human malaria parasite, *Plasmodium falciparum*. Biol. Lett..

[11] Bomblies A (2012). Modeling the role of rainfall patterns in seasonal malaria transmission. Climatic Change.

[12] Jones, A. *Seasonal Ensemble Prediction of Malaria in Africa*. Ph.D. thesis. http://ethos.bl.uk/ProcessSearch.do?query=479049 (2007).

[13] Martens W, Niessen LW, Rotmans J, Jetten TH, McMichael AJ (1995). Potential impact of global climate change on malaria risk. Environ. Health Perspect..

[14] Martens P (1999). Climate change and future populations at risk of malaria. Glob. Environ. Change.

[15] Ermert V, Fink AH, Morse AP, Paeth H (2012). The impact of regional climate change on malaria risk due to greenhouse forcing and land-use changes in tropical Africa. Environ. Health Perspect..

[16] Caminade C (2014). Impact of climate change on global malaria distribution. Proc. Natl Acad. Sci..

[17] Moss RH (2010). The next generation of scenarios for climate change research and assessment. Nature.

[18] Meehl GA, Boer GJ, Covey C, Latif M, Stouffer RJ (2000). The coupled model intercomparison project (cmip). Bull. Am. Meteorol. Soc..

[19] Fettweis X (2012). Estimating greenland ice sheet surface mass balance contribution to future sea level rise using the regional atmospheric climate model MAR. Cryosphere Discuss..

[20] Bakker P (2016). Fate of the atlantic meridional overturning circulation: strong decline under continued warming and greenland melting. Geophys. Res. Lett..

[21] Rignot, E., Velicogna, I., van den Broeke, M. R., Monaghan, A. & Lenaerts, J. T. Acceleration of the contribution of the greenland and antarctic ice sheets tosea level rise. *Geophys. Res. Lett*. **38**, L05503 (2011) 10.1029/2011GL046583.

[22] Gillet-Chaulet F (2012). Greenland ice sheet contribution to sea-level rise from a new-generation ice-sheet model. Cryosphere.

[23] Hemming, S. R. Heinrich events: Massive late Pleistocene detritus layers of the North Atlantic and their globalclimate imprint, *Rev. Geophys.* **42**, RG1005 (2004) 10.1029/2003RG000128.

[24] Church, J. A. et al. *Sea Level Change. Tech. Rep.* (Cambridge University Press, 2013).

[25] Swingedouw D (2009). Impact of freshwater release in the north atlantic under different climate conditions in an OAGCM. J. Clim..

[26] Defrance D (2017). Consequences of rapid ice sheet melting on the Sahelian population vulnerability. Proc. Natl Acad. Sci. USA.

[27] Warszawski L (2014). The inter-sectoral impact model intercomparison project (ISI–MIP): project framework. Proc. Natl Acad. Sci..

[28] Ryan SJ (2015). Mapping physiological suitability limits for malaria in Africa under climate change. Vector Borne Zoonotic Dis..

[29] Gething PW (2010). Climate change and the global malaria recession. Nature.

[30] Rodríguez-Fonseca B (2015). Variability and predictability of West African droughts: a review on the role of sea surface temperature anomalies. J. Clim..

[31] Nicholson SE, Dezfuli AK, Klotter D (2012). A two-century precipitation dataset for the continent of Africa. Bull. Am. Meteorol. Soc..

[32] Lee J-Y, Wang B (2014). Future change of global monsoon in the cmip5. Clim. Dyn..

[33] Stouffer RJ, Seidov D, Haupt BJ (2007). Climate response to external sources of freshwater: North atlantic versus the southern ocean. J. Clim..

[34] Mulitza S, Rühlemann C (2000). African monsoonal precipitation modulated by interhemispheric temperature gradients. Quat. Res..

[35] Kageyama M (2013). Climatic impacts of fresh water hosing under last glacial maximum conditions: a multi-model study. Climate.

[36] Liu W, Xie S-P, Liu Z, Zhu J (2017). Overlooked possibility of a collapsed atlantic meridional overturning circulation in warming climate. Sci. Adv..

[37] Caesar, L. et al. Current Atlantic Meridional Overturning Circulation weakest in last millennium. *Nat. Geosci*. **14**, 118–120 (2021) 10.1038/s41561-021-00699-z.

[38] Mulitza, S., M. et al. Sahel megadroughts triggered by glacial slowdowns of Atlantic meridional overturning, *Paleoceanography*. **23**, PA4206 (2008) 10.1029/2008PA001637.

[39] Caminade C, McIntyre KM, Jones AE (2019). Impact of recent and future climate change on vector-borne diseases. Ann. N. Y. Acad. Sci..

[40] Charney JG (1975). Dynamics of deserts and drought in the Sahel. Q. J. R. Meteorol. Soc..

[41] Zeng N, Neelin JD, Lau K-M, Tucker CJ (1999). Enhancement of interdecadal climate variability in the Sahel by vegetation interaction. Science.

[42] Smith M (2020). Incorporating hydrology into climate suitability models changes projections of malaria transmission in africa. Nat. Commun..

[43] Hoshen MB, Morse AP (2004). A weather-driven model of malaria transmission. Malar. J..

[44] Tompkins AM, Ermert V (2013). A regional-scale, high resolution dynamical malaria model that accounts for population density, climate and surface hydrology. Malar. J..

[45] Craig MH, Snow R, le Sueur D (1999). A climate-based distribution model of malaria transmission in sub-Saharan Africa. Parasitol. Today.

[46] Van Lieshout M, Kovats R, Livermore M, Martens P (2004). Climate change and malaria: analysis of the SRES climate and socio-economic scenarios. Glob. Environ. Change.

[47] Liu W (2014). African origin of the malaria parasite *Plasmodium vivax*. Nat. Commun..

[48] Ermert V, Fink AH, Jones AE, Morse AP (2011). Development of a new version of the Liverpool Malaria Model. II. calibration and validation for West Africa. Malar. J..

[49] Leedale J (2016). Projecting malaria hazard from climate change in eastern Africa using large ensembles to estimate uncertainty. Geospatial Health.

[50] Laneri K (2010). Forcing versus feedback: epidemic malaria and monsoon rains in northwest India. PLoS Comput. Biol..

[51] Linard C, Tatem AJ (2012). Large-scale spatial population databases in infectious disease research. Int. J. Health Geogr..

[52] Tompkins A, Thomson M (2018). Uncertainty in malaria simulations due to initial condition, climate and malaria model parameter settings investigated using a constrained genetic algorithm. PLoS ONE.

[53] Hay SI (2009). A world malaria map: *Plasmodium falciparum* endemicity in 2007. PLoS Med..

[54] Weiss DJ (2019). Mapping the global prevalence, incidence, and mortality of Plasmodium falciparum, 2000–17: a spatial and temporal modelling study. Lancet.

[55] Tompkins AM, Larsen L, McCreesh N, Taylor D. To what extent does climate explain variations in reported malaria cases in early 20th century Uganda? *Geospat Health*. **11**, 407 (2016) 10.4081/gh.2016.407. 10.4081/gh.2016.407 27063740

[56] Dalrymple U, Mappin B, Gething PW (2015). Malaria mapping: understanding the global endemicity of falciparum and vivax malaria. BMC Med..

[57] Dufresne J-L (2013). Climate change projections using the IPSL-CM5 earth system model: from CMIP3 to CMIP5. Clim. Dyn..

[58] Tayler K, Stouffer R, Meehl G (2012). An overview of CMIP5 and the experimental design. Bull. Am. Meteorol. Soc..

[59] Hourdin F (2013). Impact of the LMDZ atmospheric grid configuration on the climate and sensitivity of the IPSL-CM5A coupled model. Clim. Dyn..

[60] Krinner, G., N. et al. A dynamic global vegetation model for studies of the coupled atmosphere-biosphere system. *Global Biogeochem. Cycles*. **19**, GB1015 (2005) 10.1029/2003GB002199.

[61] Madec G (2008) NEMO ocean engine. Note du Pole de modélisation, Institut Pierre-Simon Laplace (IPSL), France, No 27 ISSN No 1288–1619.

[62] Aumont, O., & Bopp, L. Globalizing results from ocean in situ iron fertilization studies. *Global Biogeochem. Cycles.* **20**, GB2017 (2006) 10.1029/2005GB002591.

[63] Fichefet T, Maqueda MM (1997). Sensitivity of a global sea ice model to the treatment of ice thermodynamics and dynamics. J. Geophys. Res.: Oceans.

[64] Valcke S (2006). Oasis3 user guide (prism_2-5). PRISM Support Initiat. Rep..

[65] Lefevre F, Brasseur G, Folkins I, Smith A, Simon P (1994). Chemistry of the 1991–1992 stratospheric winter: Three-dimensional model simulations. J. Geophys. Res.: Atmosph..

[66] Hurtt GC (2011). Harmonization of land-use scenarios for the period 1500–2100: 600 years of global gridded annual land-use transitions, wood harvest, and resulting secondary lands. Climatic Change.

[67] Lean, J., Rottman, G., Harder, J. & Kopp, G. Sorce contributions to new understanding of global change and solar variability. In *The Solar Radiation and Climate Experiment (SORCE)* 27–53 (Springer, 2005).

[68] Richter I, Xie S-P (2008). On the origin of equatorial atlantic biases in coupled general circulation models. Clim. Dyn..

[69] Michelangeli, P.-A., Vrac, M. & Loukos, H. Probabilistic downscaling approaches: application to wind cumulative distribution functions. *Geophys. Res. Lett.* **36** (2009).

[70] Vrac M, Friederichs P (2015). Multivariate-intervariable, spatial, and temporal-bias correction. J. Clim..

[71] Famien, A. M. et al. A bias-corrected CMIP5 dataset for Africa using the CDF-t method – a contribution to agricultural impact studies. *Earth Syst. Dynam.* **9**, 313–338 (2018) 10.5194/esd-9-313-2018.

[72] Dee DP (2011). The era-interim reanalysis: Configuration and performance of the data assimilation system. Q. J. R. Meteorol. Soc..

