# Supplementary material for: Impact of an accelerated melting of Greenland on malaria distribution over Africa
Source: Nat Commun. 2021 Jun 25;12:3971. doi: 10.1038/s41467-021-24134-4 (PMC8233338; doi:10.1038/s41467-021-24134-4)
Supplement: Supplementary file 1 — Supplementary Information: Impact of an accelerated melting of Greenland on malaria distribution over Africa [file 41467_2021_24134_MOESM1_ESM.pdf]

Impact of an accelerated melting of Greenland on malaria distribution over Africa

Alizée Chemison[1*], Gilles Ramstein[1], Adrian M. Tompkins[2], Dimitri Defrance[3], Guigone Camus[1], Margaux Charra[1], Cyril Caminade[4,5]

May 2021

[1] Laboratoire des Sciences du Climat et de l'Environnnment (LSCE), CEA, Gif-sur-Yvette, France
[2] Abdus Salam International Centre for Theoretical Physics (ICTP), Trieste, Italy
[3] The Climate Data factory, Paris, France
[4] Department of Livestock and One Health, Institute of Infection, Veterinary and Ecological Sciences, University of Liverpool, UK
[5] Health Protection Research Unit in Emerging and Zoonotic Infections, University of Liverpool, UK

*Contact : alizee.chemison@lsce.ipsl.fr
These authors jointly supervised this work : Gilles Ramstein, Cyril Caminade

Supplementary Information

Supplementary note 1: MMMs sensitivity to climatic parameters

Malaria models vary in their formulation, threshold values and applications. Thus, although the input climate data are identical, the mean malaria state differs substantially between the models, although we find that the sign of the response to future climate changes is consistent across all models, lending confidence to the conclusions from our analysis. The Supplementary figure 1 shows the relationship between rainfall, temperature and malaria transmission risk. The MIASMA model, which tends to overestimate LTS, simulates a maximum LTS when rainfall reaches a minimum of 200 mm.month^{-1}, if the temperature is sufficient (Supplementary Fig. 1e). Even with low temperatures (between 15°C and 18°C) and relatively low rainfall (between 10 and 100 mm.month^{-1}), MIASMA simulates LTS values ranging between 3 and 5 months (Supplementary Fig. 1e). For the MARA model LTS is about 1 month and with the LMM_R$_0$ model there is no transmission (Supplementary Fig. 1c-d). The LMM model has a prevalence threshold, preventing it from reaching very high values as it is the case for the VECTRI model, which has values exceeding 80% when rainfall is greater than 300 mm.month^{-1} and temperatures between 25°C and 30°C (Supplementary Fig. 1a-b).These differences in model sensitivity to rainfall and temperature explain the different scores presented in validation section (Fig. 1).

The evolution of malaria transmission between the 2010s and 2040s according to the RCP8.5 and ICE1m scenarios are both presented in figure 2 and 3 for the LMM and MARA models and on Supplementary Figure 2 and 3 for the VECTRI, LMM_R$_0$, and MIASMA models.

The additional effect of the ice-sheet melting is shown in Supplementary Figure 4. The additional effect is presented in a time series separated into four geographical areas (see Methods). The trends are similar to those described in the results section. There is an increased risk of malaria in the East African highlands for both RCP8.5 and ICE1m scenarios (Supplementary Fig. 2-3). There is also a decrease in malaria transmission risk over the Sahel region, for the LMM_R$_0$ model (Supplementary Fig. 2d-3d) and over the northern Sahel with the VECTRI model (Supplementary Fig. 2b-3b). The VECTRI model shows a strong increase in malaria risk over most of the African continent for both RCP8.5 (Supplementary Fig. 2b) and ICE1m (Supplementary Fig. 3b).

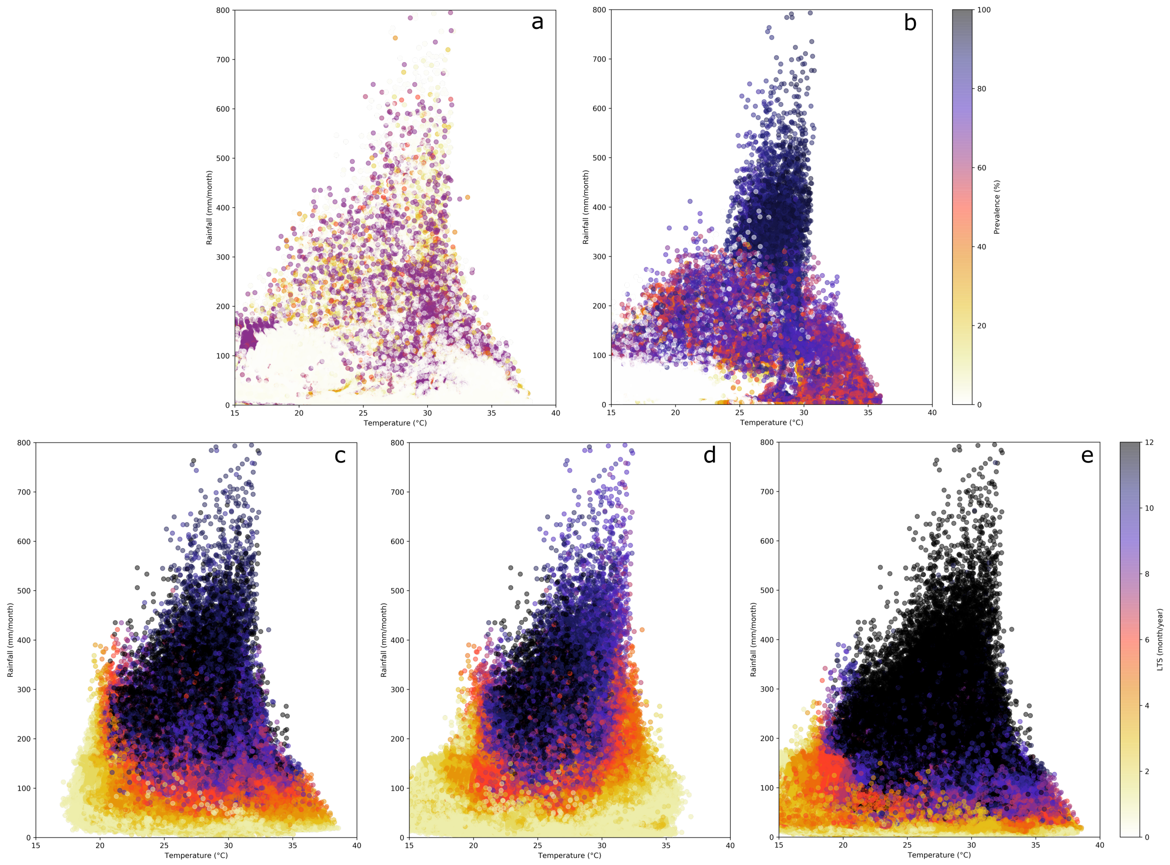

Supplementary Figure 1. Relationship between rainfall, temperature and malaria transmission risk. a-b) Prevalence (%) as a function of rainfall (mm.month^{-1}) and temperature (°C) for a) LMM and b) VECTRI. c-d-e) Length of the Transmission Season (LTS, in months.year^{-1}), for c) LMM_R$_0$, d) MARA and e) MIASMA, as a function of rainfall and temperature. The relationship is based on annual averages for all spatial points in Africa from 2000 to 2099 for the RCP8.5 simulation. Values equal to zero are not considered in the analysis.

The RCP8.5 simulation shows a decrease in LTS between the 2040s and 2010s over West Africa (Fig. 2b-d). This decrease is mostly related to an increase in temperature, as rainfall changes are close to zero (Fig. 2f-h). The relative difference between the ICE1m and RCP8.5 simulations is systematically negative over West Africa (Supplementary Fig. 4.1.a). By adding a rapid ice-sheet melting signal (ICE1m), the decrease is accentuated over West Africa with respect to the standard RCP8.5 scenario (Supplementary Fig. 4.1.a). This signal is consistent across all MMMs. The temperature increase is more moderate in ICE1m with respect to RCP8.5 (Fig. 4a). The response of MMMs over West Africa is mostly driven by accentuated drought conditions in the ICE1m experiment (Fig. 4b and Supplementary Fig. 4.1.b).

The simulated increase in malaria risk between the 2040s and the 2010s is mostly associated with both increases in temperature and rainfall for RCP8.5 over East Africa (Fig. 2). By contrast, simulated malaria risk is lower in ICE1m with respect to RCP8.5 (Supplementary Fig. 4.2). The additional release of freshwater tends to moderate the simulated increase in malaria transmission shown by RCP8.5 over East Africa. In contrast, there is a more moderate increase in simulated temperature and a drying simulated by the ICE1m simulation (Fig. 3f-h and Supplementary Fig. 4.2). However, when comparing the curves on Supplementary Figure 4.2, it would still appear that it is the changes in rainfall that lead to changes in the potential risk of malaria transmission. However, there is still a lengthening of the malaria transmission season in ICE1m over East Africa, related to more favourable temperatures conditions (Fig. 3.2).

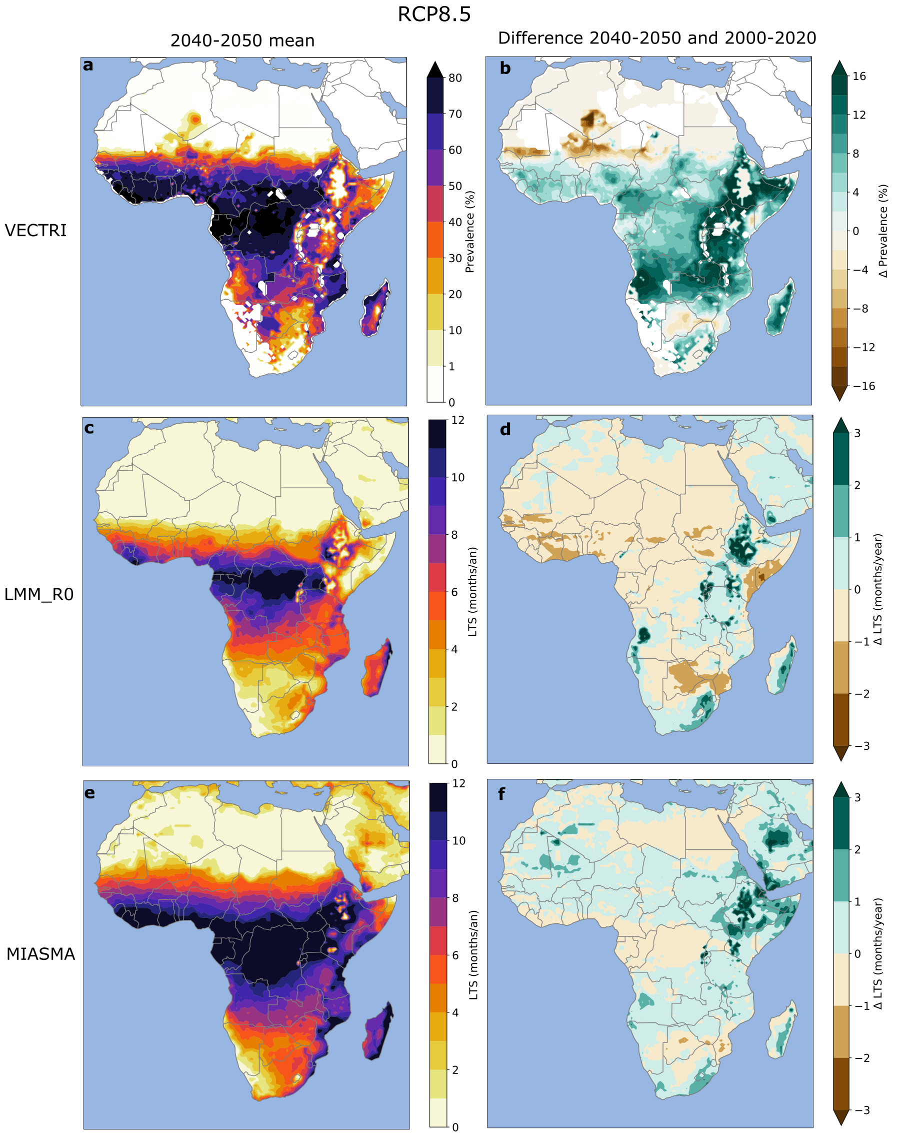

Supplementary Figure 2. Mean and future changes in malaria risk based on the RCP8.5 scenario. The left-hand columns (a-c-e) depict mean patterns for the 2040s and the right-hand columns (b-d-f) show future differences between the 2040s and 2010s. a-b) malaria prevalence (%) simulated by the VECTRI, c-d) LTS (in months.year^{-1}) based on the LMM_R_0 model, e-f) LTS (in months.year^{-1}) based on the MIASMA model.

The difference between the ICE1m and RCP8.5 simulations reaches about +15% for LMM from 2045 onwards over the coasts of Central Africa (Supplementary Fig. 4.3.a). For all other MMMs, the LTS difference is weaker but remains positive and ranges between 0 and 10% except for the VECTRI model for which differences are close to zero. These changes are correlated with decreasing temperatures while changes in precipitations are almost nil over Central (Supplementary Fig. 4.3).

The difference between ICE1m and RCP8.5 is the largest over southern Africa (Supplementary Fig. 4.4.a). This difference reaches more than 150% for simulated prevalence by LMM and more than 75% for simulated LTS by MARA and LMM_R_0 at the end of 21st century. MIASMA and VECTRI simulate a more moderate increase but it still reaches 50% at the end of the century. Differences between ICE1m and RCP8.5 are shown from 2030 onwards (Supplementary Fig. 4.4.a). These large future increases in simulated malaria risk over southern Africa are mostly related to very low malaria risk simulated during the early century baseline period (Fig. 2a-c). Therefore, the inclusion of an

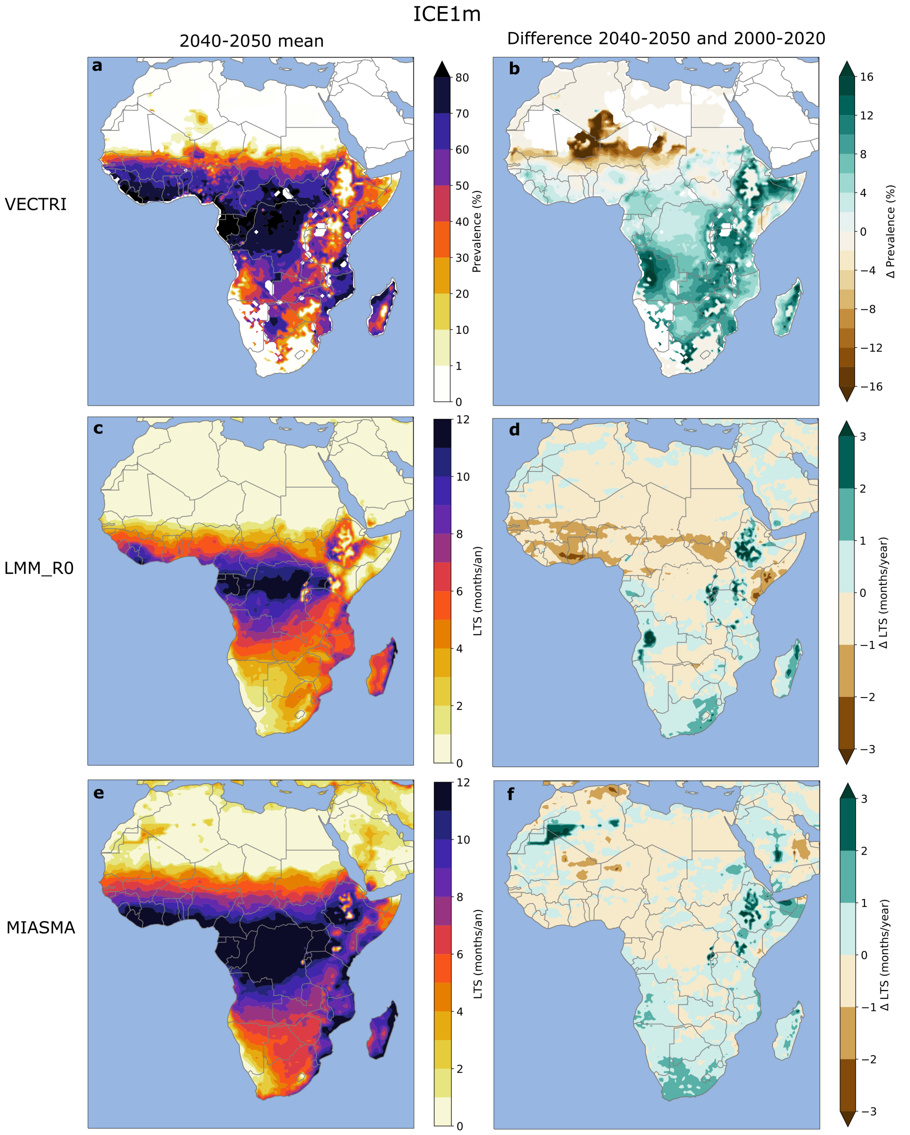

Supplementary Figure 3. Mean and future changes in malaria risk based on the ICE1m experiment. The left-hand columns (a-c-e) depict mean patterns for 2040s and the right-hand columns (b-d-f) show future differences between the periods 2040s and 2010s. a-b) malaria prevalence (%) simulated by the VECTRI model, c-d) LTS (in months.year^{-1}) based on the LMM_R$_0$ model, e-f) LTS (in months.year^{-1}) based on the MIASMA model.

additional rapid ice sheet melting, tends to cause a 2-3 months increase in LTS and a 20% increase in simulated prevalence over southern Africa (Supplementary Fig. 4.4.a). Such a signal is consistent with a large increase in simulated precipitation over the region which is consistent with a southward shift of the rain-belt over the African continent (Fig. 4b and Supplementary Fig. 4.4.b).

Regions where there is a decrease in simulated malaria transmission for the ICE1m simulation (Fig. 5 and Supplementary Fig. 4.1.a-2.a) correspond to areas where there is a clear decrease in simulated precipitation (Fig. 4b and Supplementary Fig. 4.1.b-2.b). Areas with higher LTS and prevalence values (Fig. 5 and Supplementary Fig. 4.3.a-4.a) roughly correspond to regions experiencing a significant increase in precipitation (Fig. 4b and Supplementary Fig. 4.3.b-4.b). Similar analyses were carried out for the ICE0.5m, 1.5m and 3m scenarios. Similar trends are shown for the 1.5m and 3m experiment, while the signal is noisy for the ICE0.5m experiment.

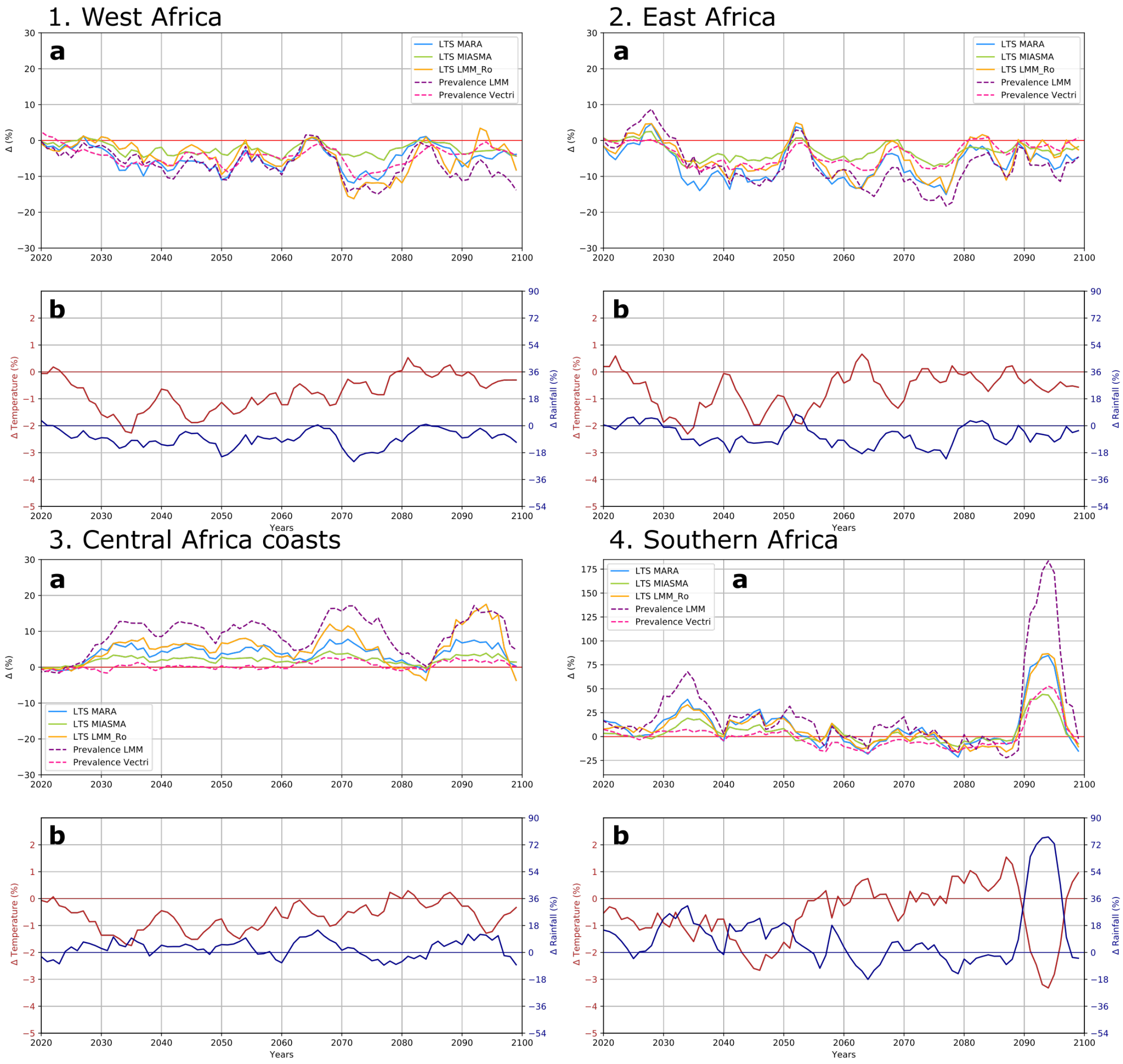

Supplementary Figure 4. Relative difference in malaria transmission risk between the ICE1m and RCP8.5 per African regions. Plots 1, 2, 3 and 4 respectively represent West Africa (red box), the coasts of Central Africa (blue box), East Africa (yellow box) and southern Africa (green box), see methods for a precise definition of the geographical domains. a) Variations in malaria prevalence and LTS transmission risk according to the different mathematical malaria models. b) Associated relative differences in temperature and rainfall. A 6-years running average was applied to all time series and relative changes are shown in percentages. Note that the y-axis values are different for the southern African region, where simulated changes are much higher than for the other regions (4.a)

Although there are differences in sensitivity between the models and therefore different quantitative results, the simulated trends are identical in their sign, providing confidence in the broad conclusions drawn.

Supplementary note 2: Endemicity classes

The differences in LTS presented in figure 5 can lead to changes in endemicity classes. The following analysis shows that there is no change in endemicity classes for more than 80% of spatial point (Supplementary Table 1). When there is a change, it is usually by at most one class (Supplementary Fig. 5). These changes take place at the edges of regions of the same class, suggesting that the response in the models is dominated by temperature changes rather than those of precipitation, as the former is spatially rather uniform and would lead to changes in endemicity class to occur at the class boundaries. Instead the spatially heterogeneous precipitation signals could in theory class changes within regions of a given class. Decreases are occurring over the northern half of Africa, particularly over the Sahel and East African plateau regions, consistently with results presented in figure 5. Increases are shown over the southern half of Africa, for: Namibia, Botswana and South Africa (Supplementary Fig. 5).

a. LMM_R_0			
No change in endemicity classes	Occurrence of malaria	Malaria Disappearance	Changes inter endemicity classes
89,9%	1,9%	2,0%	6.2%
21569 points	461 points	478 points	1492 points

b. MARA			
No change in endemicity classes	Occurrence of malaria	Malaria Disappearance	Changes inter endemicity classes
90,3%	0,9%	1,3%	7,5%
21674 points	225 points	310 points	1791 points

c. MIASMA			
No change in endemicity classes	Occurrence of malaria	Malaria Disappearance	Changes inter endemicity classes
81,1%	2,8%	3,7%	12,4%
19475 points	663 points	891 points	2971 points

Supplementary Table 1. Distribution of endemicity class changes between the ICE1m and RCP8.5 simulations for the period 2040-2050, a) for LMM_R_0, b) MARA and c) MIASMA.

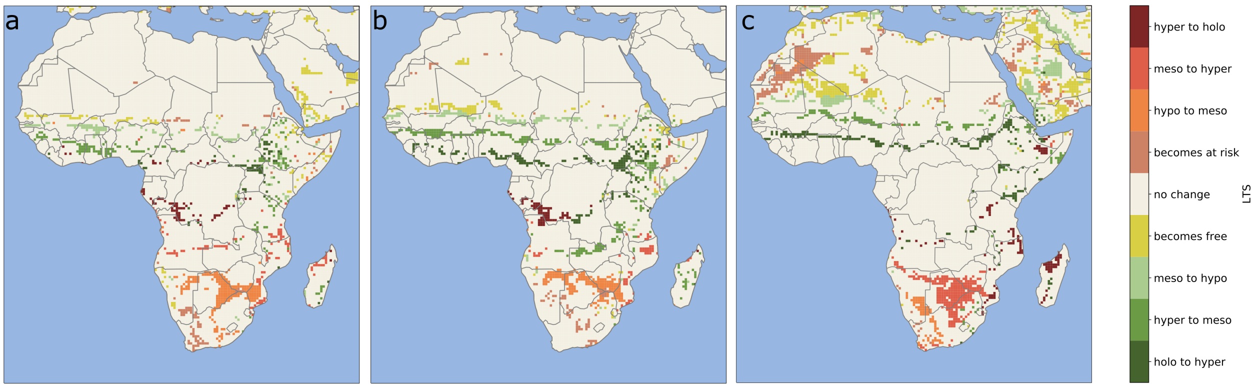

Supplementary Figure 5. Change in endemicity classes between the ICE1m and RCP8.5 scenarios for the period 2040-2050 for a) LMM_R$_0$, b) MARA and c) MIASMA. The five categories are free, hypoendemic (hypo), mesoendemic (meso), hyperendemic (hyper) and holoendemic (holo). "Becomes free" corresponds to the transition from an endemic class to malaria free class. "Becomes at risk" corresponds to the transition from malaria free class to an endemic category. See methods for the definition of thresholds for the different classes.

Supplementary note 3: MMMs sensitivity to different ice-melting scenario

Supplementary figures 6, 7, 8 and 9 show the sensitivity of the MMMs to the different scenarios of ice-sheet melting used as inputs. The temporal evolution is shown on Supplementary figure 6, the additional effect of the release of fresh water is shown on Supplementary figure 7, transient changes in climatic parameters are shown on Supplementary figure 8 and transient changes in malaria transmission are shown on Supplementary figure 9.

The largest the amount of freshwater released, the smallest the temperature increases between the 2040s and the 2010s period (Supplementary Fig. 6.c-g-k-o-s). This signal is associated with a significant drought over West Africa, East Africa and most of Central Africa and a large increase in rainfall over southern Africa (Supplementary Fig. 6.d-h-l-p-t). These differences in rainfall and temperature are consistent with simulated changes in malaria transmission (Supplementary Fig. 6-7). It can also be noted that the ICE1m and 1.5m simulations provide very similar results (Supplementary Fig. 7). On the other hand, inter-annual variability in simulated rainfall and malaria parameters is very large (Supplementary Fig. 8). It is noteworthy that the differences in malaria risk are only negative or positive for ICE1m, 1.5, 3m but the simulation ICE0.5m shows non-significant changes, this is the case for West Africa, and Central Africa (Supplementary Fig. 9). Nevertheless, the responses vary greatly depending on the region studied, the selected year and the MMM.

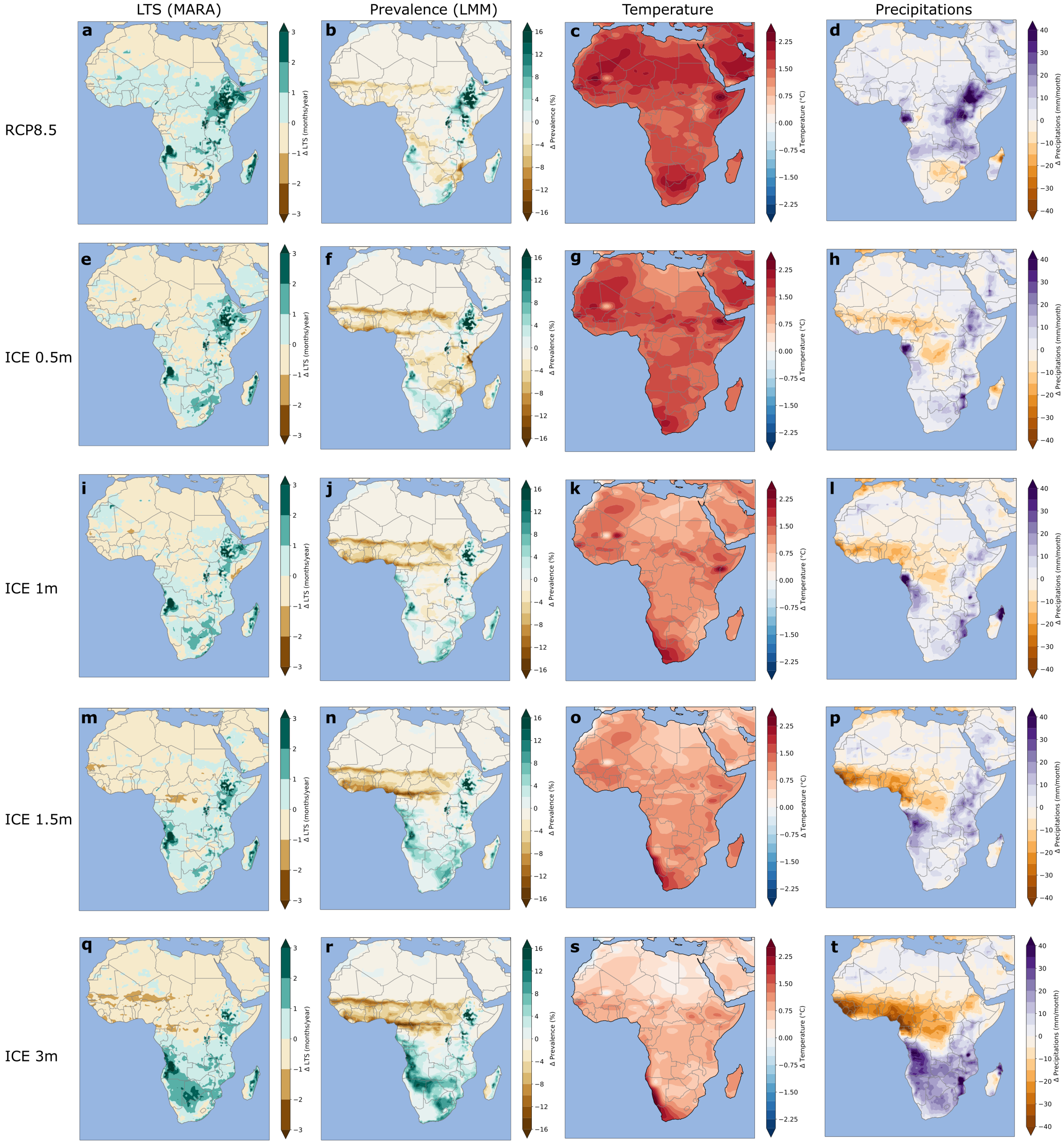

Supplementary Figure 6. Temporal difference between the periods 2040s and 2010s. a-b-c-d) RCP8.5, e-f-g-h) ICE0.5m, i-j-k-l) ICE1m, m-n-o-p) ICE1.5m and q-r-s-t) ICE3m simulation. a-e-l-m-q) LTS (months.year^{-1}) for the MARA model, b-f-j-n-r) prevalence (%) with the LMM model, c-g-k-o-s) temperature (°C) and d-h-l-f-t) precipitation (mm.month^{-1}).

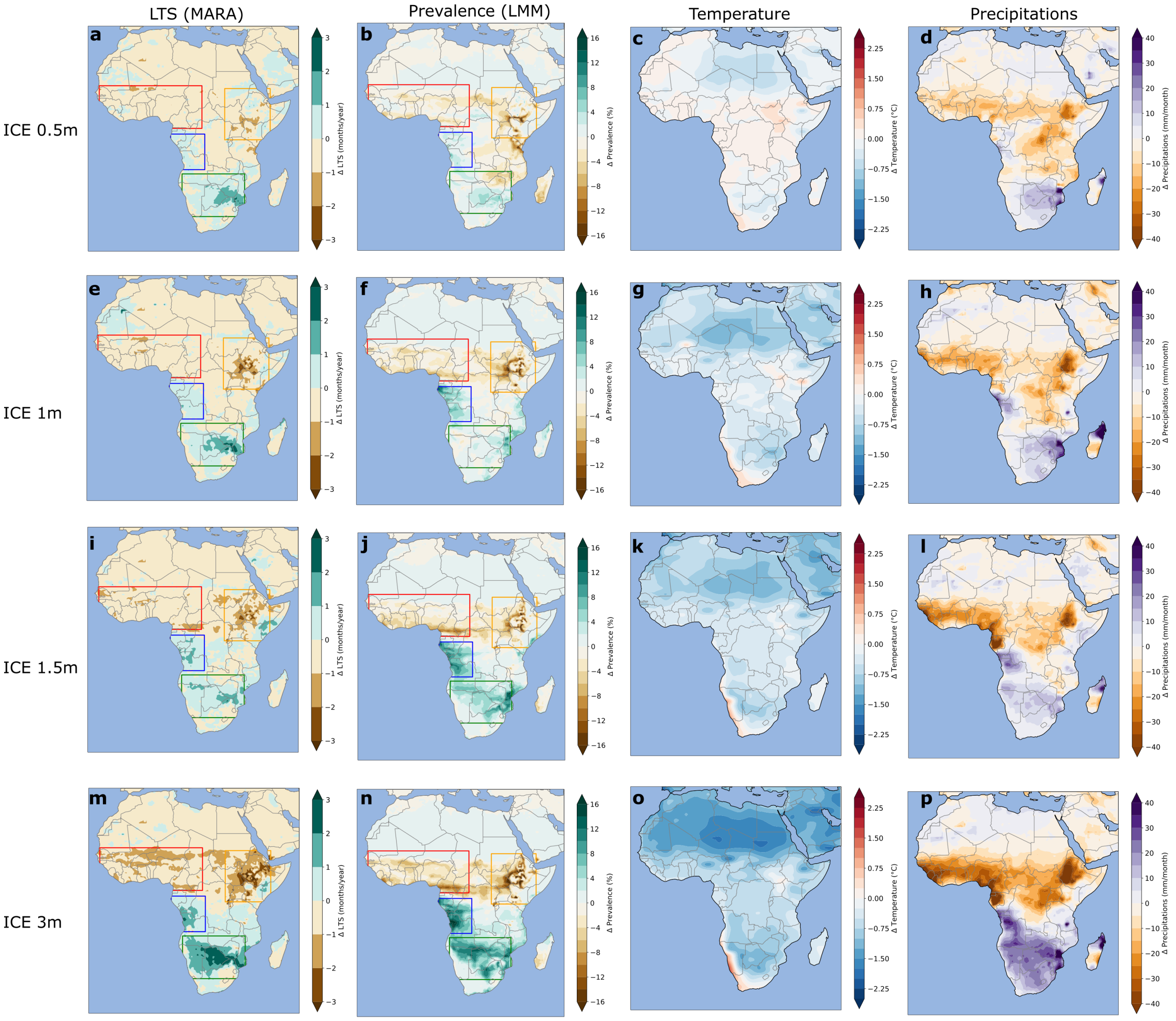

Supplementary Figure 7. Difference between the ICEXm simulations and the standard RCP8.5 simulation for the period 2040-2050. a-b-c-d) ICE0.5m, e-f-g-h) ICE1m, i-j-k-l) ICE1.5m, m-n-o-p) ICE3m. a-e-i-m) difference in LTS (months.year^{-1}) for MARA, b-f-j-n) prevalence (%) for LMM c-g-k-o) temperature (°C), d-h-l-p) precipitation (mm.month^{-1}).

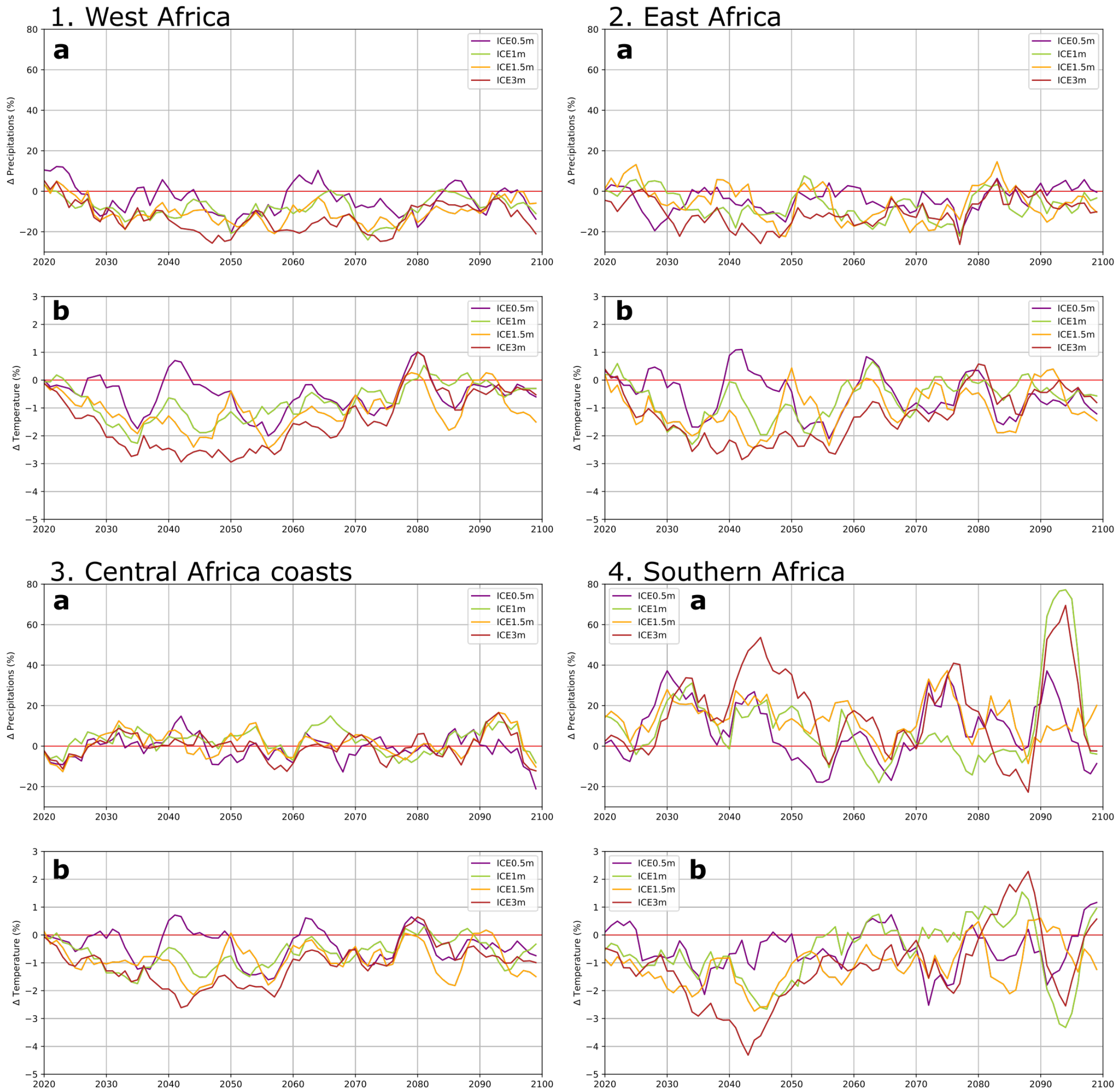

Supplementary Figure 8. Climate difference between the ICEXm and the standard RCP8.5 simulation per African regions for a) rainfall and b) temperature. Plots 1, 2, 3 and 4 respectively represent West Africa (red box), the coasts of Central Africa (blue box), East Africa (yellow box) and southern Africa (green box), see methods for a precise definition of the geographical domains. A 6-years running average was applied to all time series and relative changes are shown in percentages.

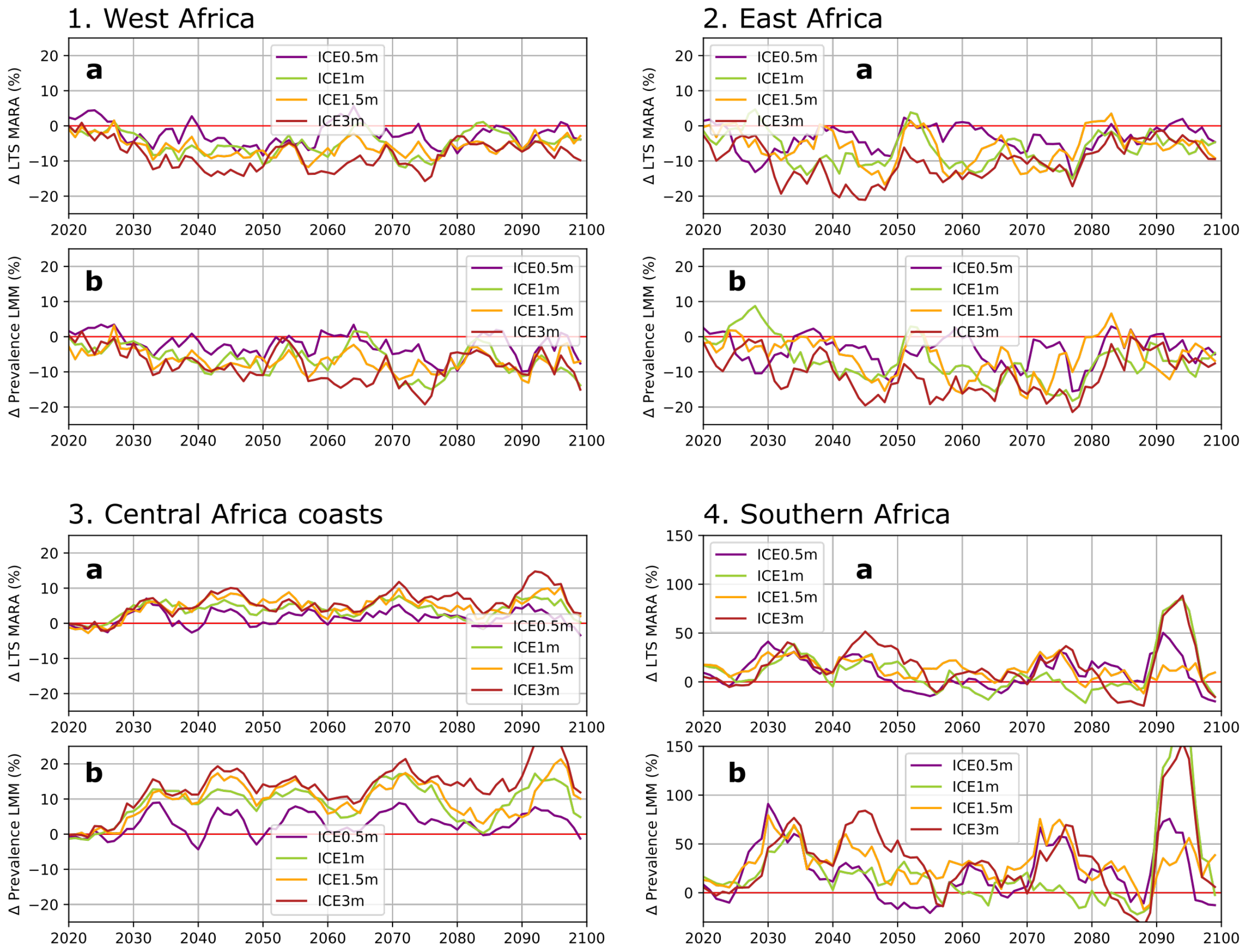

Supplementary Figure 9. Difference in malaria transmission risk between the ICEXm and the standard RCP8.5 simulation per African regions, according to mathematical malaria models, a) MARA, b) LMM. Plots 1, 2, 3 and 4 respectively represent West Africa (red box), the coasts of Central Africa (blue box), East Africa (yellow box) and southern Africa (green box), see methods for a precise definition of the geographical domains. A 6-years running average was applied to all time series and relative changes are shown in percentages. Note that the y-axis values are different for the southern African region, where simulated changes are much higher than for the other regions (4.a).
