# Supplementary material for: Impact of an accelerated melting of Greenland on malaria distribution over Africa
Source: Nat Commun. 2021 Jun 25;12:3971. doi: 10.1038/s41467-021-24134-4 (PMC8233338; doi:10.1038/s41467-021-24134-4)
Supplement: Supplementary file 2 — Reporting Summary [file 41467_2021_24134_MOESM2_ESM.pdf]

nature research

Corresponding author(s): Alizée Chemison
Last updated by author(s): May 17, 2021

# Reporting Summary

Nature Research wishes to improve the reproducibility of the work that we publish. This form provides structure for consistency and transparency in reporting. For further information on Nature Research policies, see our Editorial Policies and the Editorial Policy Checklist.

## Statistics

For all statistical analyses, confirm that the following items are present in the figure legend, table legend, main text, or Methods section.

| n/a | Confirmed | |
|---|---|---|
| x | | The exact sample size ($n$) for each experimental group/condition, given as a discrete number and unit of measurement |
| x | | A statement on whether measurements were taken from distinct samples or whether the same sample was measured repeatedly |
| | x | The statistical test(s) used AND whether they are one- or two-sided<br>*Only common tests should be described solely by name; describe more complex techniques in the Methods section.* |
| x | | A description of all covariates tested |
| x | | A description of any assumptions or corrections, such as tests of normality and adjustment for multiple comparisons |
| x | | A full description of the statistical parameters including central tendency (e.g. means) or other basic estimates (e.g. regression coefficient) AND variation (e.g. standard deviation) or associated estimates of uncertainty (e.g. confidence intervals) |
| x | | For null hypothesis testing, the test statistic (e.g. $F$, $t$, $r$) with confidence intervals, effect sizes, degrees of freedom and $P$ value noted<br>*Give P values as exact values whenever suitable.* |
| x | | For Bayesian analysis, information on the choice of priors and Markov chain Monte Carlo settings |
| x | | For hierarchical and complex designs, identification of the appropriate level for tests and full reporting of outcomes |
| | x | Estimates of effect sizes (e.g. Cohen's $d$, Pearson's $r$), indicating how they were calculated |

*Our web collection on statistics for biologists contains articles on many of the points above.*

## Software and code

Policy information about availability of computer code

| | |
|---|---|
| Data collection | N/A. No software was used for data collection. |
| Data analysis | The code of the VECTRI model is available in open-source at the following link: http://users.ictp.it/~tompkins/vectri/<br>For other models, please contact the creators of these codes: Anne Jones (anne.jones@ibm.com) or Andy Morse (a.p.morse@liverpool.ac.uk) for LMM and LMM_Ro - M. Craig for MARA (craigm@mrc.ac.za) and Pim Martens for MIASMA (p.martens@icis.unimaas.nl)<br><br>IPSL climate model simulations were derived from the work of Defrance et al. (https://doi.org/10.1073/pnas.1619358114).<br>The annual averages as well as the maps and statistical tests have been carried out using python scripts, python version 3. |

For manuscripts utilizing custom algorithms or software that are central to the research but not yet described in published literature, software must be made available to editors and reviewers. We strongly encourage code deposition in a community repository (e.g. GitHub). See the Nature Research guidelines for submitting code & software for further information.

## Data

Policy information about availability of data

All manuscripts must include a data availability statement. This statement should provide the following information, where applicable:

- Accession codes, unique identifiers, or web links for publicly available datasets
- A list of figures that have associated raw data
- A description of any restrictions on data availability

All model inputs and outputs that support the findings of this study are available at https://www.doi.org/10.17605/OSF.IO/RHKPB that should allow reproducibility of the main figures. The MAP data can be accessed by following this link: https://malariaatlas.org/explorer/# , they are available for use under the Creative Commons Attribution 3.0 Unported license. The Lysenko and Semashko data has been digitized from the paper of Gething et al., 2010

# Field-specific reporting

Please select the one below that is the best fit for your research. If you are not sure, read the appropriate sections before making your selection.

☐ Life sciences ☐ Behavioural & social sciences ☒ Ecological, evolutionary & environmental sciences

For a reference copy of the document with all sections, see nature.com/documents/nr-reporting-summary-flat.pdf

# Ecological, evolutionary & environmental sciences study design

All studies must disclose on these points even when the disclosure is negative.

| | |
|---|---|
| Study description | This study shows the impact of an accelerated melting of the Greenland ice sheet, simulated by the IPSL climate model on malaria transmission in Africa simulated by five mathematical malaria models. Our results show that a rapid sea-ice melting at high latitudes induce an increase of malaria transmission risk over southern Africa. |
| Research sample | The research sample consists of monthly malaria risk of transmission for plasmodium falciparum in Africa.<br>Malaria simulations were carried out for the period 1951-2099, at 0.5 degrees resolution for Africa for MARA, MIASMA and LMM_R0 and for the period of 2000-2099 for VECTRI. All variables were derived at monthly time scale. |
| Sampling strategy | The land surface of Africa was sampled at a spatial resolution of 0.5 degrees. |
| Data collection | All model inputs and outputs for the RCP8.5 and ICE1m are available on OSF at https://www.doi.org/10.17605/OSF.IO/RHKPB |
| Timing and spatial scale | Spatial scale : data on a 0.5 x 0.5 degree grid<br>Timing : 01/01/1951 to 31/12/2099 monthly timestep for MARA, MIASMA and LMM_R0, daily timestep for VECTRI and LMM. For VECTRI the timing is different: 01/01/2000 to 31/12/2099. |
| Data exclusions | None |
| Reproducibility | Due to the large amount of data and malaria model copyright, we provide all model inputs and outputs at https://www.doi.org/10.17605/OSF.IO/RHKPB that should allow are available reproducibility of the main figures. |
| Randomization | N/A. This is not an experimental study. |
| Blinding | N/A. This is not an experimental study. |

Did the study involve field work? ☐ Yes ☒ No

# Reporting for specific materials, systems and methods

We require information from authors about some types of materials, experimental systems and methods used in many studies. Here, indicate whether each material, system or method listed is relevant to your study. If you are not sure if a list item applies to your research, read the appropriate section before selecting a response.

## Materials & experimental systems

| n/a | Involved in the study |
|---|---|
| ☒ | ☐ Antibodies |
| ☒ | ☐ Eukaryotic cell lines |
| ☒ | ☐ Palaeontology and archaeology |
| ☒ | ☐ Animals and other organisms |
| ☒ | ☐ Human research participants |
| ☒ | ☐ Clinical data |
| ☒ | ☐ Dual use research of concern |

## Methods

| n/a | Involved in the study |
|---|---|
| ☒ | ☐ ChIP-seq |
| ☒ | ☐ Flow cytometry |
| ☒ | ☐ MRI-based neuroimaging |
